# Design and Investigation of New Water-Soluble Forms of α-Tocopherol with Antioxidant and Antiglycation Activity Using Amphiphilic Copolymers of *N*-Vinylpyrrolidone

**DOI:** 10.3390/pharmaceutics15051388

**Published:** 2023-04-30

**Authors:** Yuliya V. Soldatova, Irina I. Faingold, Darya A. Poletaeva, Alexei V. Kozlov, Nina S. Emel’yanova, Igor I. Khodos, Dmitry A. Chernyaev, Svetlana V. Kurmaz

**Affiliations:** 1 Federal Research Center of Problems of Chemical Physics and Medicinal Chemistry, Russian Academy of Sciences, Academician Semenov av., 1, 142432 Chernogolovka, Russia; ifaingold@mail.ru (I.I.F.); daryazhokhova@gmail.com (D.A.P.); n_emel@mail.ru (N.S.E.); chernyayevda@icp.ac.ru (D.A.C.); skurmaz@icp.ac.ru (S.V.K.); 2 Institute of Microelectronics Technology and High-Purity Materials, Russian Academy of Sciences, Institutskaya Street, 6, 142432 Chernogolovka, Russia; khodos@iptm.ru

**Keywords:** *N*-vinylpyrrolidone, (di)methacrylates, amphiphilic copolymers, α-tocopherol, encapsulation, lipoxidation, advanced glycation end products, glycation

## Abstract

Water-soluble forms of α-tocopherol (TP) as an effective antioxidant were obtained by encapsulating it into nanoparticles (NPs) of amphiphilic copolymers of *N*-vinylpyrrolidone with triethylene glycol dimethacrylate (CPL1-TP) and *N*-vinylpyrrolidone with hexyl methacrylate and triethylene glycol dimethacrylate (CPL2-TP) synthesized by radical copolymerization in toluene. The hydrodynamic radii of NPs loaded with TP (3.7 wt% per copolymers) were typically ca. 50 or 80 nm depending on copolymer composition, media, and temperature. Characterization of NPs was accomplished by transmission electron microscopy (TEM), IR-, and ^1^H NMR spectroscopy. Quantum chemical modeling showed that TP molecules are capable to form hydrogen bonds with donor groups of the copolymer units. High antioxidant activity of both obtained forms of TP has been found by the thiobarbituric acid reactive species and chemiluminescence assays. CPL1-TP and CPL2-TP effectively inhibited the process of spontaneous lipid peroxidation as well as α-tocopherol itself. The IC_50_ values of luminol chemiluminescence inhibition were determined. Antiglycation activity against vesperlysine and pentosidine-like AGEs of TP water-soluble forms was shown. The developed NPs of TP are promising as materials with antioxidant and antiglycation activity and can be used in various biomedical applications.

## 1. Introduction

Fat-soluble α-tocopherol (TP) is the most prevalent and effective form of vitamin E [1], which is a chain-breaking antioxidant in human plasma and tissues [2,3]. α-Tocopherol effectively intercepts free radicals of unsaturated lipids by transferring the phenolic H-atom to propagating peroxyl or alkoxyl radicals with a constant rate that is higher than the rate of chain propagation, giving a non-radical lipid product and the α-tocopheroxyl radical (TP•). The α-tocopheroxyl radicals immediately react with other free radicals or each other, forming a non-radical species [4].

Glycation is a non-enzymatic reaction between free amino groups of amino acids and aldehyde or ketone groups of reducing sugars leading to the development of advanced glycation end products (AGEs) [5]. This process participates in the complications linked with disorders such as diabetes, cancer, and cardiovascular diseases. α-Tocopherol has a good inhibitory effect on the process of non-enzymatic protein glycation in vitro [6,7,8] and in vivo [9,10,11,12]. Vitamin E supplementation can reduce blood glycated hemoglobin levels in rats and diabetic patients [13,14,15].

Non-enzymatic glycation is closely related to lipid peroxidation (LPO). Free radical formation (and other byproducts of lipid peroxidation, ex., malone dialdehyde (MDA)) participates in non-enzymatic reactions with glucose and radical scavengers are known to be effective glycation inhibitors. The mechanism through which α-tocopherol reduces protein glycation was shown to be inhibition of lipid peroxidation, which contributes to the glycation of proteins [6]. Vitamin E also diminishes protein glycation by lowering the formation of the LPO product MDA, linked to AGE synthesis [16]. Furthermore, α-tocopherol in vitro inhibits potent formation of carboxymethyllysine from glycated human serum albumin [17], a reaction of glycated proteins that depends upon reactive oxygen species (ROS).

Vitamin E deficiency occurs due to common fat malabsorption syndromes (Crohn’s disease, cholestatic disease) [18]. Abetalipoproteinemia and chylomicron retention disease are rare recessive forms of hypobetalipoproteinemia that is characterized by intestinal lipid malabsorption and severe deficiency of vitamin E [19]. Studies have shown that using water-soluble forms of α-tocopherol can significantly increase its absorption in the intestine to normal plasma concentrations [20]. In other cases, not only in pharmacological applications, stabilized water-soluble versions of TP are desirable to improve its bioavailability [21,22].

TP is generally used as a functional unit to form polymeric nanocarriers in drug delivery systems [23,24] or in wound dressings [25] because of its great antioxidant activity. Some water-soluble forms of TP (such as D-α-tocopheryl-polyethylene-glycol-succinate—vitamin E TPGS) are employed also as solubilizing agent for lipophilic drugs (retinoic acid, paclitaxel, and resveratrol) [26,27]. In addition to pharmacological use, TP is widely used in the cosmetology and food industries [28]. However, there are significant limitations in the pharmaceutical, food, and cosmetic industry applications due to TP’s insolubility in water and instability upon exposure to light, temperature, and oxygen [29,30,31].

Approaches to improve the solubility of lipophilic molecules in water and raise their potential for different applications include use as a co-solvent, in solid dispersion, chemical modification, or through a supramolecular complex [21,32,33,34,35].

Polymeric carriers of the micellar type based on the amphiphilic block copolymers and liposomes with encapsulated hydrophobic drugs demonstrate high efficiency [36,37,38]. Our studies have shown that amphiphilic copolymers based on *N*-vinylpyrrolidone (VP) with branches in polymer chains proved to be promising as platforms for lipophilic compounds (zinc tetraphenylporphyrinate, iron nitrosyl complexes, fullerenes, methyl pheophorbide a, etc.) [39,40,41,42,43] with low cytotoxicity and ability to penetrate cells in vitro to deliver the active agent [39,44].

The preparation of water-soluble forms of TP based on *N*-vinylpyrrolidone with branches in polymer chains and studying their properties is a promising direction for their biomedical (and broader) application.

Nanosized systems of various biologically active compounds (BAC) based on amphiphilic copolymers of *N*-vinylpyrrolidone with (di)methacrylates can be obtained by a plain method [45]. It includes the hydrophobic BAC encapsulation into nanoparticles of VP amphiphilic copolymers at concentrations around the critical concentration of aggregation in isopropyl alcohol and following dissolution of polymer films in aqueous medium. The drug association with the copolymers proceeds through trap sites within the polymer matrix and penetration of drug molecules into their hollows, forming complexes such as the “guest–host” type or drug adsorption on the nanoparticles’ surface. Here, we propose amphiphilic copolymers and terpolymers of *N*-vinylpyrrolidone branched with triethylene glycol dimethacrylate (TEGDM) as carriers of TP to increase its water solubility and membranotropic properties.

The aim of the present study is to prepare water-soluble forms of TP by its encapsulation in nanoparticles of amphiphilic copolymers of *N*-vinylpyrrolidone with triethylene glycol dimethacrylate (CPL1-TP) and *N*-vinylpyrrolidone with hexyl methacrylate and triethylene glycol dimethacrylate (CPL2-TP), to characterize them by various methods including quantum chemical modeling of intermolecular interaction between TP and copolymers and study their antioxidant and antiglycation activity in vitro.

## 2. Materials and Methods

### 2.1. Chemicals and Materials

N-vinylpyrrolidone (99%, VP, Alfa Aesar) was distilled in vacuum to remove the NaOH inhibitor. Triethylene glycol dimethacrylate (95%, TEGDM, Aldrich, St. Louis, MO, USA) and hexylmethacrylate (HMA) were used without additional purification. 2,2′-Azo-bis-isobutyronitrile (AIBN) purified by recrystallization from ethanol was used as the initiator of radical polymerization. We used toluene (analytical grade) and n-hexane (reagent grade).

**Figure 1:**
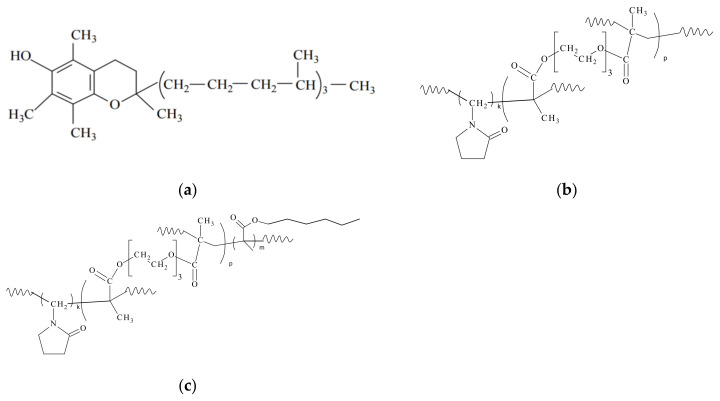
Chemical formulas of TP (**a**) and CPL1 (**b**) and CPL2 (**c**).

D-α-tocopherol (Figure 1a) was purchased from Alfa Aesar. Aminoguanidine, sodium azide, thiobarbituric acid (TBA), trichloroacetic acid (TCA), *tert*-butyl hydroperoxide (TBHP), and luminol were purchased from Sigma (St. Louis, MO, USA). Bovine serum albumin (BSA) (fraction V) and D-glucose were purchased from Life Science (USA). Folin’s reagent was purchased from Applichem (Darmstadt, Germany).

### 2.2. Synthesis of the Copolymers

The amphiphilic copolymers (CPL1 and CPL2) with the chemical structures (Figure 1b,c) were produced by the radical copolymerization in toluene from monomer mixture [VP]:[HMA]:[TEGDM] of molar composition of 100:0:2 and 100:2:2 according to the procedure [44]. The synthesis of the copolymers was carried out in a three-necked flask equipped with a reflux condenser and thermometer and the Ar bubbles flowed continuously for 2 h at 80 °C. All components of the reaction mixture were introduced simultaneously. The content of reagents in the solvent was ~20 wt%. The concentration of the initiator in the solution was 0.02 mol·L^−1^. After completion of the reaction, toluene-soluble products were obtained; the reaction mixture was transparent and homogeneous. The isolation of the copolymers from the toluene solution was carried out by precipitation with the tenfold excess of n-hexane as a precipitant. The precipitates were filtered in a Buchner funnel and dried from solvent/precipitant to constant weight in air and in vacuum. The CPL1 and CPL2 yield was 94.5 and 95%, resp.

### 2.3. The Method of TP Copolymer Compositions’ Formation

To study the antioxidant and antiglycation activity in vitro, the copolymer compositions were obtained with TP content of ~3.7%. Encapsulation of drug into copolymer particles was carried out using solutions of the copolymers and TP in isopropyl alcohol through their mutual association. For this, TP solution was added dropwise to the copolymer solutions under constant stirring. All mixtures remained clear and homogeneous. After drying with isopropyl alcohol, the polymer films containing TP were dissolved in water or aqueous neutral buffer solution (pH 6.8–7.0 or 7.2–7.4) and were analyzed by different methods. To prepare the CPL1-TP composition, 100 mL of a copolymer solution in IPA (7 mg/mL) and 18.3 mL of TP in IPA (1.4 mg/mL) were used. To prepare the CPL2-TP composition, 100 mL of a solution of the CPL2 copolymer in IPA (7 mg/mL) and 18 mL of TP in IPA (1.4 mg/mL) were used. PBS contains: 1.37 mM NaCl, 2.68 mM KCl, 4.29 mM Na_2_HPO_4_, 1.47 mM KH_2_PO_4_. For physicochemical studies, NPs with various TP loadings were produced. For this purpose, the volume of TP solution added to the copolymer solution in isopropyl alcohol was varied; encapsulating conditions, reagent concentrations, and TP content (%) per copolymer are given in Supplementary Materials, Tables S1–S3.

### 2.4. Elemental Analysis

The nitrogen content in copolymers was determined from the elemental analysis data obtained with Vario MICRO cube (Elementar Analysensysteme GmbH, Langenselbold, Germany). The nitrogen content in CPL1 and CPL2 was 11.2 and 11.0%, respectively.

### 2.5. Size Exclusion Chromatography

The absolute molar masses of the copolymers were determined by size exclusion chromatography using a Waters liquid chromatograph (2 columns PS-gel, 5 μm, MIXED-C, 300 × 7.5 mm) (Waters Corp, Milford, MA, USA)) supplied with a refractive index detector and a multi-angle light scattering detector WYATT DAWN HELEOS II (Wyatt Technology, Santa Barbara, CA, USA), λ = 658 nm. *N*-methylpyrrolidone with the addition of lithium chloride (1 wt%) was used as an eluent to prevent macromolecules’ aggregation in a polar solvent. The measurement was carried out at 70 °C; the elution rate was 1 mL min^−1^. The dn/dc value was defined from multi-angle light scattering detector data. All copolymer solutions (10–20 mg mL^−1^) were previously filtered (pore diameter 0.2 μm). Number average of molecular weight of the copolymer was calculated from light scattering data using Astra software 5.3.2.20 (Wyatt Technology, Goleta, CA, USA).

### 2.6. Dynamic Light Scattering

The hydrodynamic radii, R_h_, of studied copolymers and copolymer-TP structures were determined using dynamic light scattering (DLS). Solutions were previously filtered (pore diameter 0.45 μm), and the vials filled with the solution were thermostated for 20 min. A temperature range for measurements was 20–55 °C. DLS measurements were performed using a Photocor Compact instrument (Photocor Ltd., Moscow, Russia) with a laser diode (654 nm). Solutions of copolymers and copolymer-TP structures were evaluated at a detection angle of 90 °C. Data analysis was performed using the DynaLS software, version 2.8.3 (Dr. Alexander A Goldin, Alango Ltd., Tirat Carmel, Israel). The size distribution curves were recorded. The hydrodynamic radii Rh of the TP loaded copolymer particles were calculated using the Einstein–Stokes equation

D = kT/6πηR,(1)

where D is the diffusion coefficient, k is the Boltzmann constant, T is the absolute temperature (K), and η is the viscosity of the medium, in which the dispersed particles are suspended.

### 2.7. Electronic Absorption Spectroscopy

The absorption spectra of aqueous solutions of the copolymers loaded with TP were recorded using the SPEKS SPP-705-1 spectrometer. The dependence 1/(A − A_0_) was used to determine the K_ef_ value by Connors’ method [46]; A and A_0_ are the optical densities at the maximum of the absorption band at a wavelength of 290 nm of encapsulated TP and copolymer, respectively, on the value of 1/[TP]. The intersection and the slope of linear dependence were obtained. Their ratio correlated to the value of the effective binding constant. It was concluded that the interaction between the ligand L and the substrate S is 1:1; so a single complex SL (1:1) was formed. It was also concluded that the sites are independent and all species obeyed the Beer–Lambert law. At chosen wavelength, the molar absorptivity of the substrate (ε_S_) and molar absorptivity of the complex (ε_11_) were different.

### 2.8. TEM Study of CPL2 and CPL1-TP, CPL2-TP

TEM images of copolymers and CPL1-TP, CPL2-TP were obtained from aqueous solutions using the JEM-2100 microscope operating at 200 kV. For studying in the microscope, a drop of an aqueous suspension was applied to a copper grid covered with a carbon film and dried.

### 2.9. Quantum Chemical Calculations

The optimized geometry of TP molecule, monomers’ sites, and the dimer structures of its H-complexes TP with the copolymer site were obtained by quantum chemical modeling performed using Gaussian09/tpssh/6-31G*. As starting geometries of dimer structures, at the first stage of calculations, all possible options were tested based on the presence of functional groups capable of forming hydrogen bonds. There were no imaginary oscillation frequencies in the calculations; all optimized structures corresponded to the potential energy minimum. Wave functions were analyzed using the QTAIM method and the AIMALL software package (version 10.05.04) [47]. The wave functions of the structures were calculated in the same way. The energies of intermolecular bonds were calculated by the following formula [48]:

E_a−b_ ≈ 1/2ν_e_(r), (2)

where E_a−b_ is the A − B bond energy and ν_e_(r) is the potential energy density at the bond critical point A − B.

### 2.10. Tissue Preparation

All manipulations on animals were carried out in accordance with the EU Directive 2010/63/EU. Hybrid BDF1 mice, 6 months old, were decapitated. For the thiobarbituric acid reactive substances assay, each brain was briskly removed, placed in liquid nitrogen, and stored at −80 °C. For the luminol chemiluminescence assay, the recently prepared brains were defrosted and homogenized using a WisdWiseTis HG-15D homogenizer for 2 min in 0.1 M Tris-HCl, pH 7.4. Protein concentrations were determined using the Lowry method [49].

### 2.11. Luminol Chemiluminescence Assay

Measurements were carried out using a 1250 Luminometer (LKBWallac, Turku, Finland) [50]. The reaction mixture contained mouse brain homogenate (protein concentration 0.1 mg/mL) in 0.1 M Tris-HCl (Sigma-Aldrich, St. Louis, MO, USA), pH 7.4, luminol (Sigma-Aldrich) (0.05 mM), *tert*-butylhydroperoxide (TBHP) (Sigma-Aldrich) (0.073 M) and tested compounds. The probe was placed in a light-proof luminometer chamber, and the background signal was recorded. The reaction proceeded at 37 °C. The control was set to give a signal of 10 mV using a built-in standard. The free radical content in mouse brain homogenate was determined by the change in the light sum, which is the area under the kinetic curve of luminescence intensity for the entire luminescence time. All studied compounds were examined at least in triplicate. The IC50 values were calculated.

### 2.12. TBARS Assay

The procedure was carried out in accordance with the known method [51]. Mouse brain homogenate in phosphate buffer (pH 7.2) was thermostated at 37 °C for 30 min with the tested compound. The reaction was stopped by addition of 17% (*w*/*v*) trichloroacetic acid (TCA) (Sigma-Aldrich). After centrifugation at 1300× *g* for 20 min, 0.8% (*w*/*v*) thiobarbituric acid (TBA) (Sigma-Aldrich) was added to 1 mL of supernatant, heated for 30 min at 95 °C. The thiobarbituric acid reactive substances (TBARS) assay quantifies the levels of MDA and other minor aldehyde species through their reaction with TBA. The optical density was measured at 532 nm using an Agilent Cary 60 UV–vis spectrophotometer (Agilent, Santa Clara, CA, USA). TBARs concentration was calculated using an extinction coefficient of 1.56 × 10^5^ M^−1^cm^−1^. All compounds were examined at least in triplicate. IC_50_ values were calculated.

### 2.13. The Antiglycation Assay

The antiglycation assay was performed using the methods [52] with slight modifications. The probe contained bovine serum albumin (BSA) (4 mg/mL), D-glucose (0.4 M) in Na-phosphate buffer (pH = 7.4, sodium azide content 0.02%), and tested compound. The final assay volume was 1 mL. Aminoguanidine was used as a positive control [53]. The reaction mixture was incubated at 60 °C for 48 h. The reaction was stopped by adding 100% (*w*/*v*) TCA. The TCA-added mixture was kept at 4 °C for 10 min before centrifugation at 135,000 rpm using the Ohaus Frontier 5515R centrifuge (4 min, 4 °C). The precipitate was redissolved with 1 mL alkaline PBS (pH = 10).

Content of glycated BSA was determined using spectrofluorometer Cary Eclipse (Agilent Technologies, Santa Clara, CA, USA). Wavelengths used: λ_exc/em_ = 370/440 nm for vesperlysines-like AGE and λ_exc/em_ = 335/385 for pentosidine-like AGE [54]. The percentage of AGE formation was calculated as follows:

*I* (%) = (*F*(_*BSA* + *D-glucose* + *test compound*_) − *F*_(*BSA* + *test compound*)_)/(*F*_(*BSA* + *D-glucose*)_ − *F*_(*BSA*)_) × 100 (3)

IC_50_ values were calculated.

### 2.14. Statistical Analysis

All analyses were performed using the statistical software package Origin 9.1 and GraphPad Prism 8. All in vitro assays were carried out in triplicate, and values were shown as mean ± standard error of the mean (SEM). The significance between groups was analyzed by using the Student’s *t*-test. *p* values < 0.05 were considered statistically significant.

## 3. Results and Discussion

### 3.1. The VP Copolymer Parameters and Properties

The VP copolymers (chemical structures are shown on Figure 1b,c) were obtained by radical copolymerization in toluene. Bifunctional and monofunctional methacrylic comonomers such as TEGDM and HMA-like MMA [55] were more reactive in radical copolymerization than VP. This was confirmed by kinetics study of copolymerization in a broad range of VPs and dimethacrylate conversions and by analysis of the monomer composition of the resulting copolymers by IR spectroscopy and isothermal calorimetry [56]. The rate of VP conversion was much lower than that of dimethacrylate. All radicals added more active monomers at the initial stage of copolymerization, and polymer chains were formed enriched with (di)methacrylate unites. Their distribution in growing polymer chains was statistical and bulky alkyl substitution of HMA limitation of intermolecular cross-linking led to insoluble macrogel formation. However, nanogels and microgels as internally cross-linked particles of small size can be formed. The copolymer structure formed at the initial stages of the reaction contained “pendant” C=C bonds of TEGDM units, through which growing PVP chains are attached [56]. This was supported by the second peak on the copolymerization curve of VP and EGDM, which is the simplest representative of a series of dimethacrylates [56].

**Figure 2:**
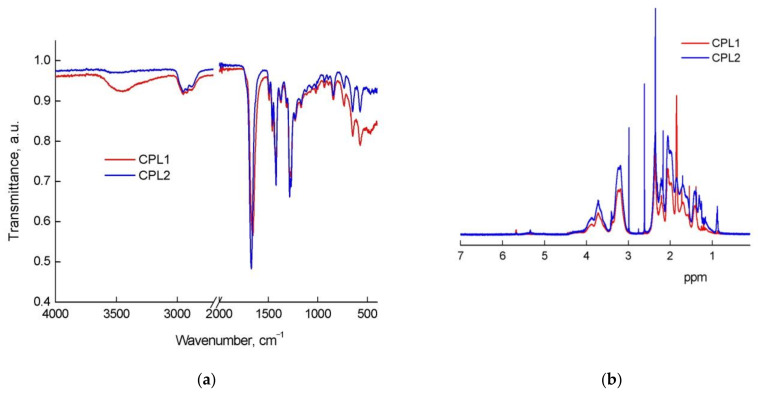
IR (**a**) and ^1^H NMR spectra (**b**) of CPL1 and CPL2 in deuterated chloroform (6 mg/mL). ^1^H NMR spectra are normalized to the maximum peak of the solvent; the reference signal is the solvent at 7.26 ppm.

CPL1 and CPL2 were identified by IR spectroscopy (Figure 2a) over absorption bands corresponded to the stretching of the C=O units of methacrylate and VP at ~1720 cm^−1^ (shoulder) and ~1665 cm^−1^ (intense band), respectively. The absorption peak at ~1380 cm^−1^ is caused by C–N–C fragment in the VP units. The peaks at 3000–2800 cm^−1^ are attributed to symmetric and asymmetric C–H stretching of –CH_3_– and –CH_2_– groups in TEGDM, HMA, and VP units. In the IR spectrum of CPL1 in the region of 3600–3000 cm^−1^, a broad absorption band is observed, which is characteristic of the stretching vibrations of the OH groups of adsorbed water hydrogen-bonded to the C=O groups of the VP units of the copolymers [57]. The relative water content is lower in hydrophobic CPL2. Moreover, the position of C=O bond involved in its formation in the CPL2 spectrum corresponds to the band of 1670 cm^−1^, while it is 1655 cm^−1^ in CPL1. Thus, copolymers of various amphiphilicity differ significantly in their affinity for polar water molecules and their adsorption.

The ^1^H NMR spectra (Figure 2b) provide evidence that copolymers had signals of VP and TEGDM units. Thus, two groups of signals related to the VP units are observed in spectra. The first group has the signals of NCH_α_ protons in the polymer chain and CH_2_C=O fragments of pyrrolidone at δ 3.0–4.0. The second group is represented by the signals of CH_2_ protons in the polymer chain and C–CH_2_–C and NCH_2_ fragments of pyrrolidone at δ 1.4–2.4 ppm. Wide signal at 4.1 ppm corresponds to the hydrogen atoms in the –CH_2_–CH_2_– fragment of the TEGDM units. At δ ~5.6 ppm weak signals of protons of “pendant” C=C bonds of TEGDM are observed. The proton signals of the CH_3_ group of the TEGDM units were observed in the copolymers spectrum at ~0.9 ppm. The intensity of proton signals in the CH_3_ and CH_2_ groups increased in the CPL2 spectrum in the region of ~1, 1.3 ppm due to the presence of HMA units in the copolymer chains.

The physicochemical characteristics of the VP copolymers are shown in Table 1. Their monomer compositions were calculated from elemental analysis data. The (di)methacrylates units content was below 5 mol.%. The copolymers have similar absolute molecular weight, M_w,_ and low PD (M_w_/M_n_) values. Near the critical aggregation concentration (CAC) of the CPL1 in water, the solution included scattering centers of different hydrodynamic radius (Table 1). It can be assumed that they corresponded to individual macromolecules as monomolecular micelles of R_h_ ca. 4 nm with a low-polarity polar and nucleus shell and aggregates (multimolecular micelles) of R_h_ ca. 77 nm due to their self-assembly [58]. The CAC values were obtained from the DLS data using the semilogarithmic dependence of light scattering intensity on the copolymer concentration as in [41]. They can be approximated by linear functions and their intersection corresponded to the CAC copolymers in water. The CAC copolymers value depends on chemical composition and CPL2 with hydrophobic HMA units are most prone to aggregate formation. The aggregates with R_h_ ca. 90 nm present only in CPL2 water solution above CAC value.

**Figure 3:**
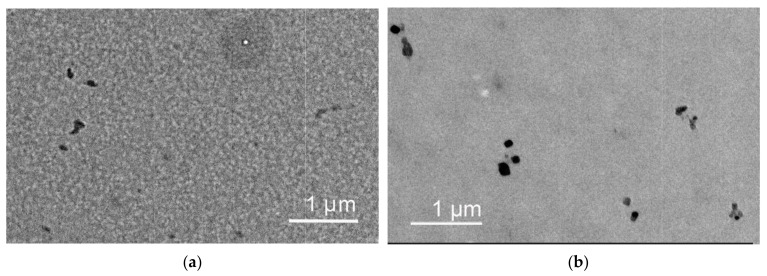
TEM images of CPL2 obtained from aqueous solutions: (**a**,**b**) the dark particles of different forms of the size up to 100 nm; (**a**) spherical particles with a contrasting white core and shell of weak contrast of the size up to 1 micron.

TEM images of dried CPL2 are shown in Figure 3. Two types of particles were found: the dark particles of different forms of the size up to 100 nm (Figure 3a,b) and spherical particles with a contrasting white core and shell of weak contrast of the size up to 1 micron (Figure 3b). The shell is formed by hydrated PVP polar chains and is typical of amphiphilic VP-TEGDM copolymers displaying data from experiment and quantum chemical modeling [57]. The second hydration shell was formed by water molecules hydrogen-bonded to each water molecule from the first hydration shell. Weakly bound water molecules evaporate during sample preparation, while water strongly bound to the polar groups of the copolymer forms the shell of the polymer particles.

### 3.2. Encapsulation TP in VP-Copolymer and Characterization of TP-NPs

Water soluble TP-NPs were prepared by a simple and efficient method of direct dissolution in two steps as described in the Materials and Methods. In the first stage, solutions of the copolymer and TP in isopropyl alcohol were used to maintain the uniformity of the medium and prevent self-association of the reagents. As a result, homogeneous and transparent mixtures were produced; after evaporation of the common solvent for the reagents at room temperature, transparent films with distributed drug in a copolymer matrix were obtained. In the second step, the dry films of the copolymers with drug content up to 14% per the copolymer were solved in PBS at room temperature, and clear or opalescent water solutions of TP-NPs were obtained.

The TP encapsulation into copolymers was performed in IPA as a thermodynamically relevant solvent for amphiphilic VP copolymers. Individual macromolecules with a hydrodynamic radius of about 4 nm were found in these solutions (Supplementary Materials, Figure S1) that reacted weakly to temperature changes from 25 to 55 °C. TP was added to the copolymer solution associate with the copolymers through the physical trap of the drug molecules within the polymer matrix and entered into their hollows, forming non-covalent guest–host complexes. Hydrophobic interactions between TP and (di)methacrylates moieties stimulate the molecules’ penetration into the nucleus of monomolecular micelles. In a water solution, these interactions are enhanced, and stable nanostructures of TP-CPL such as multimolecular micelles are formed [58]. The water solutions of CPL-TP were slightly or strong opalescent depending on the TP content per copolymer particles, and concentration in solutions and media (water or PBS).

**Figure 4:**
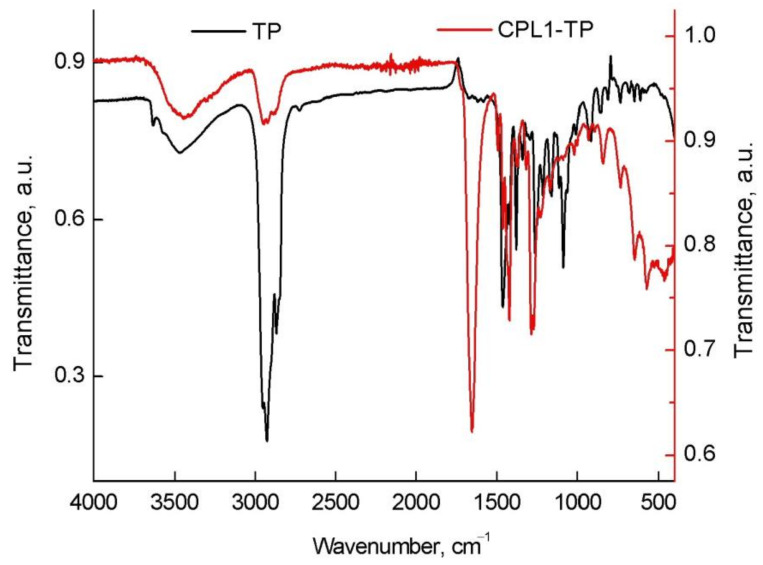
IR spectra of TP and a powder of TP-CPL1.

TP is soluble in alcohol and its absorption spectra in IPA are shown in Figure S2 (Supplementary Materials). The absorption dependence of the band at 290 nm wavelengths on TP concentration was linear and obeyed the Beer–Lambert law [59]; thus, there were not any intermolecular interactions between the components of solution. However, the hydroxyl group of TP can partake in the formation of intermolecular hydrogen bonds [60]. The formation of intermolecular H-bonds was evidenced by the wide absorption band of stretching vibrations of the OH-group at 3700–3100 cm^−1^ in its IR spectrum (Figure 4). Meantime, some of the OH groups of TP remained free, which is proved by the restricted absorption band at 3630 cm^−1^. In the range of 1610–1580 cm^−1^, stretching vibrations of the C=C bond of the aromatic nucleus are seen. The characteristic absorption bands at 1097 and 813 cm^−1^ belong to the tetrahydropyran cycle and the band at 1169 cm^−1^ concerns the tocol fragment [60]. According to [61], the TP biological activity is associated with these structural fragments.

**Figure 5:**
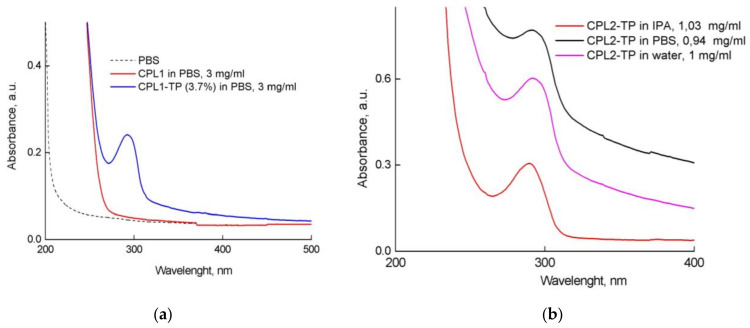
Absorption spectra of TP-CPL1 (**a**) and TP-CPL2 (**b**) in aqueous solutions (water, PBS) (cuvettes were 0.2 or 1 cm).

TP is hydrophobic and the absorption spectra of aqueous solutions in control experiments without copolymers have no band at 280–300 nm (Supplementary Materials, Figure S3). Thereby, we conclude that all TP molecules are encapsulated in the copolymer NPs. In the absorption spectra of TP-CPL1 and TP-CPL2 aqueous solutions (Figure 5), there is a TP absorption band at 292 nm due to its solubilization by amphiphilic copolymer particles, so TP solubility was increased. Its expanded and asymmetric shape is probably due to TP localization in the copolymer particles.

An important parameter of TP and a copolymer interaction in aqueous solution is the effective binding constant K_ef_ [46]. To calculate its values, we used the Connors method and dilute solutions and different volumes of TP solution (Supplementary Materials, Tables S1 and S2). Absorption spectra of aqueous buffer solution of TP encapsulated into CPL1 and CPL2 and dependence of the optical density of CPL2-TP on TP concentration in the Connors equation coordinates are presented in Figures S4 and S5 (Supplementary Materials). The effective binding constant K_ef_ of TP with CPL1 and CPL2 was equal to 0.72 × 10^4^ and 1.35 × 10^4^ M^−1^ at 22 °C, resp. Thus, both copolymers bonded to TP through intermolecular interactions, primarily the hydrogen bonding which is formed among the OH group of TP and the donor groups of the copolymers [59]. It is expected that hydrophobic interactions between the alkyl substituent of TP and alkyl substituents of HMA occur when guest molecules are localized in the low-polarity core of the CPL2 nanoparticles.

**Figure 6:**
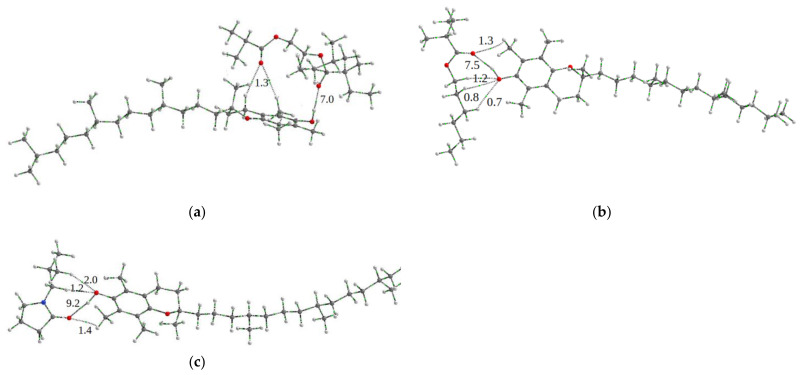
Molecular graphs of optimized geometries of TP-monomer units’ structure obtained by the QTAIM method: (**a**) TP-TEGDM; (**b**) TP-HMA; (**c**) TP-VP. Bond energies are given in kcal/mol.

Quantum chemical modeling has shown that TP molecules are able to form hydrogen bonds with all three monomeric units of copolymers through the OH group. We present here the calculated binding energies for TEGDM, and HMA of copolymers, as well as VP units, the content of which in the hydrophobic core of NPs is negligible. Figure 6 shows the TP-copolymer unit structures obtained by the QTAIM method, in which possible intermolecular bonds are shown and their energies are given, calculated by the Espinoza–Lecomte formula from the analysis of the critical points of the bonds formed. The bond energies are approximately the same.

Quantum chemical modeling [59] showed that TP molecules can form hydrogen bonds with donor groups of TEGDM-units in the hydrophobic core of the VP-TEGDM copolymer particles. The length of the hydrogen bond among the OH group of TP and the C=O group of the TEGDM unit was 1.931 Å (tpssh/6-31G*), which indicates its adequate strength. The TP orientation due to the hydrophobic brush of TEGDM proposes the possibility of attachment of a few TP molecules to this polymer moiety.

**Figure 7:**
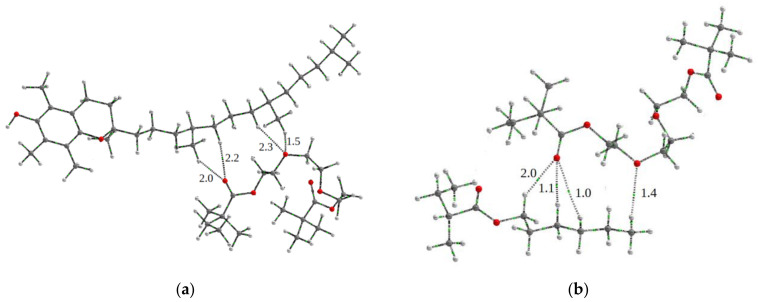
Molecular graphs of optimized structure geometries obtained by the QTAIM method: (**a**) TP-TEGDM; (**b**) HMA-TEGDM. Bond energies are given in kcal/mol.

Here, we considered the interactions between the oxygen atoms of the ether groups of both simple and ester groups, and the hydrogen atoms of the TP hydrocarbon tail, since they also contribute to intermolecular interactions (Figure 7a). Oxygen of the ester groups of TEGDM in CPL2 can be shielded by the HMA hydrocarbon tail, forming hydrogen bonds (Figure 7b). This can lead to a decrease in the contribution of hydrogen bond to the total binding energy. However, the existing intermolecular interactions are quite sufficient for the existence of a “guest–host” complex based on the CPL2.

Absorption bands of the copolymers on IR spectra of the TP-CPL1 and TP-CPL2 are shown in Figure 2a. So, in the TP-CPL1 spectrum there is an intensive absorption band of stretching vibrations of the C=O group in the lactam cycle of VP units at 1651 cm^−1^, forming the hydrogen bond with water. The C=O oscillations of the TEGDM group were presented as a shoulder at 1721 cm^−1^. The main TP absorption bands were not observed in the copolymer compositions, apparently due to its poor content.

The behavior of CPL1-TP prepared for biological tests was studied by DLS in PBS (pH 7.2–7.4) in the temperature range of 25–47 °C. Figure S6 (Supplementary Materials) shows the corresponding distribution curves of the light scattering intensity over the hydrodynamic radii of scattering centers. The TP-CPL1 solution scatters more strongly than CPL1 itself at the same concentrations in PBS, as follows from the temperature dependences of the light scattering intensity and hydrodynamic radius of the scattering centers (Supplementary Materials, Figure S7). As a consequence, the corresponding dependence I(T) lies higher in the studied temperature range. The intensity sharply increases at 40 °C and a break indicates an increase in TP-CPL1 aggregation with temperature growth. This means that the TP-NPs solution is thermosensitive, and near the copolymer LCST its turbidity is observed. The initial CPL1 was characterized by a bimodal distribution of scattering centers and responded to temperature increase also at lower values of ~33–34 °C. TP increases the hydrophobicity of these NPs and their tendency to aggregate in PBS increases. Consequently, the hydrodynamic radius of NPs increases with temperature and reaches ~90 nm (Supplementary Materials, Figure S7b).

**Figure 8:**
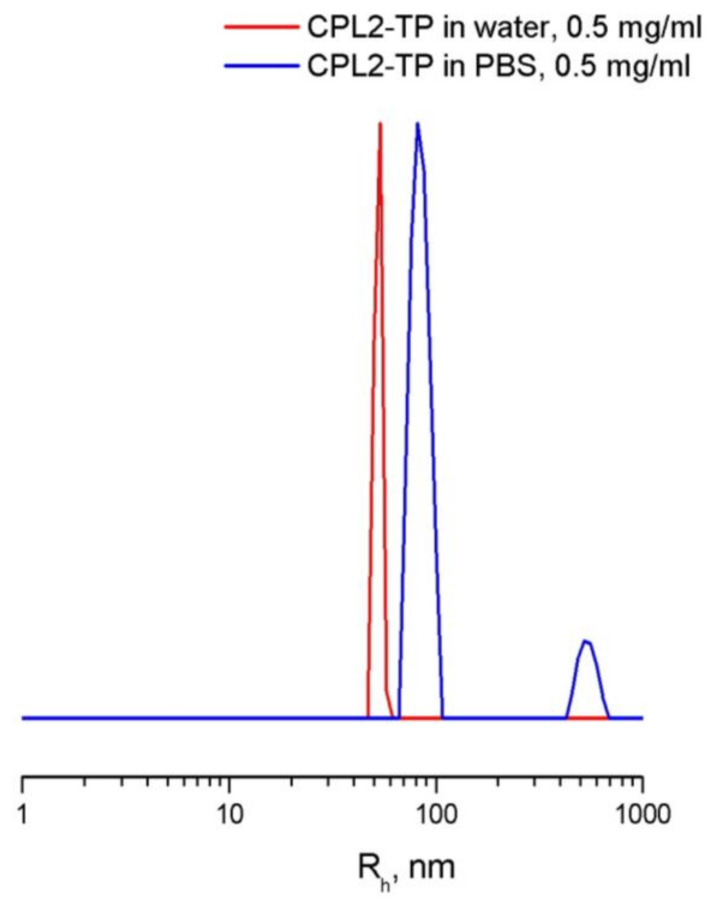
DLS curves of TP-CPL2 in water and PBS (channel range is 30–170) at 25 °C.

Figure 8 shows the DLS curves of the TP-CPL2 NPs in water and PBS. In water, the particle size distribution of light scattering intensity is unimodal, in contrast to PBS, which has larger aggregates. At the peak maximum, the Rh values in water and PSB were ~56 and 84 nm, respectively; pH has a significant effect on the sizes of scattering centers, and in water without of salts, the sizes of aggregates are smaller. As a result, water solutions are transparent, while buffer solutions are opalescent. With a temperature increase up to 50 °C, the intensity of light scattering by the aqueous buffer solution TP-CPL2 increased, and the size of the scattering centers at the maximum of the main peak reached ~100 nm. Thus, TP-CPL2 exhibited thermal sensitivity, like TP-CPL1.

**Figure 9:**
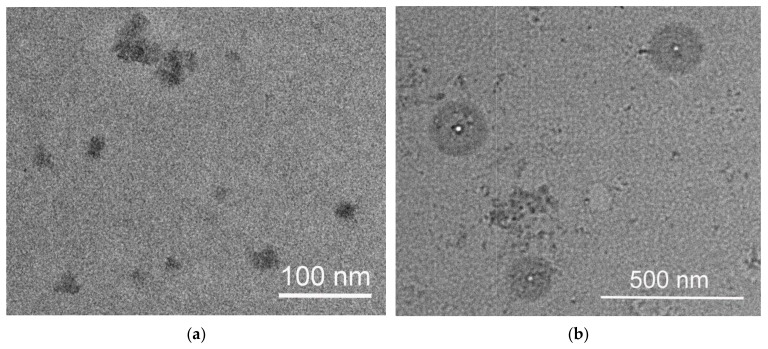

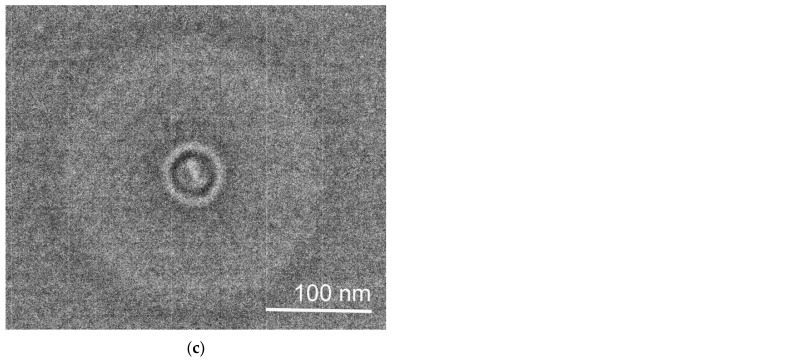
TEM images of CPL1-TP obtained from aqueous solutions: (**a**) the examples of spherical particles with a contrast core and low contrast periphery; (**b**,**c**) the particles of a different type at different magnifications.

Figure 9 shows TEM images of CPL1-TP and CPL2-TP obtained from aqueous solutions. The structure of CPL1-TP was characterized by particles usually up to 60–70 nanometers in size (Figure 9a) and spherical particles with a pronounced shell ranging in size from about 150 to 300 nm (Figure 9a,b). A multilayer morphology with different substance densities and contrasts of a particle with a diameter of about 250 nm (Figure 9c) can be seen at detailed examination. The particles can have a highly contrasting core with a diameter of several tens of nanometers.

**Figure 10:**
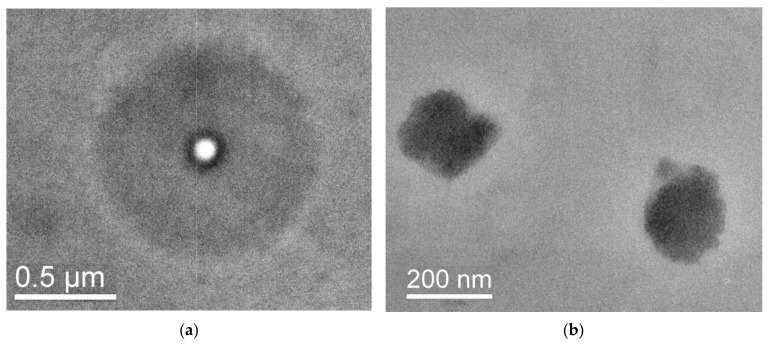

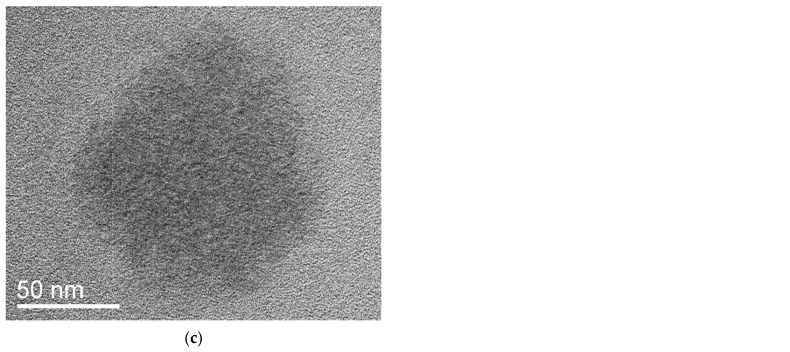
TEM images of the particles in the CPL2-TP: (**a**) spherical particle, (**b**,**c**) images of particles with uniform contrast at different magnifications.

Sample CPL2-TP was characterized by particles similar to those found in CPL1-TP. Figure 10 shows the examples of spherical particles with a contrast core and low contrast periphery (Figure 10a) and the particles of a different type (Figure 10b,c). When considering the objects, weakly contrast areas are visible; perhaps these were cavities that could be occupied by guest molecules.

The stability of CPL2-TP (Supplementary Materials, Table S3) in aqueous buffer solutions was studied by electron absorption spectroscopy. The absorbance of TP in freshly prepared solutions rises with increasing TP content in NPs (Supplementary Materials, Figure S8). The absorption spectra of the studied solutions practically did not change during the observation period, since TP was not released from NPs, and TP-CPL2 did not precipitate from solution (Supplementary Materials, Figure S8). Moreover, an aqueous solution of TP-CPL2 was exposed to temperatures of 50 and 80 °C for 3 h, but the optical density of the TP absorption band remained unchanged, which indicated the high stability of NPs. Thus, up to 14% TP per the CPL2 can be entered into copolymer particles under given conditions.

**Figure 11:**
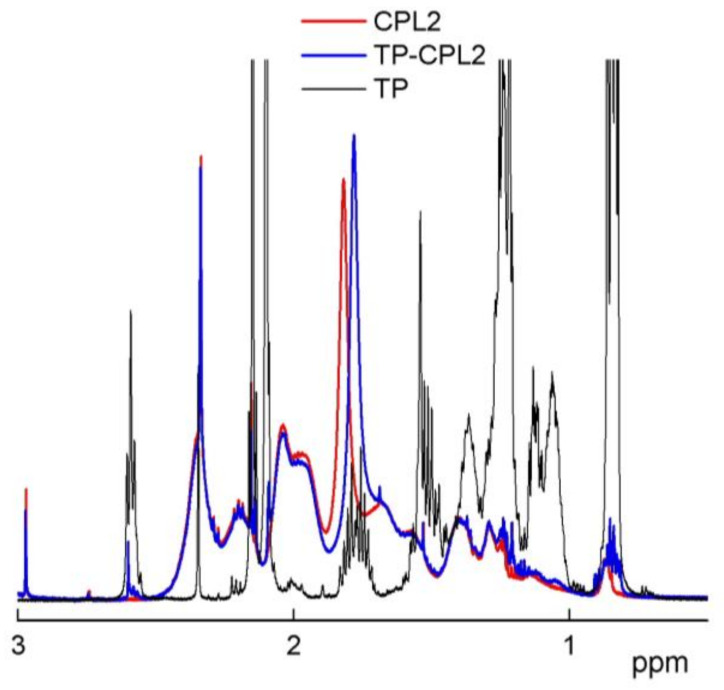
^1^H NMR spectra of TP, CPL2, and TP-CPL2 (3.7 wt%) in deuterated chloroform in the range of 0–3 ppm. Spectra normalized and calibrated for solvent signal at 7.26 ppm.

Figure 11 shows the ^1^H NMR spectrum of TP-CPL2 in deuterated chloroform in the range 0–3 ppm. The spectra of CPL2 and TP are given for comparison. In the TP-CPL2 spectrum, TP signals are visible, most of which overlap with those of the copolymer. Only the CH3 groups of the TP molecule stand apart. The shift of the CPL2 signal in the region of 1.8 ppm in the presence of TP is observed.

Thus, water-soluble forms of TP based on copolymers of different monomer composition and amphiphilicity were obtained; the maximal concentration of the soluble drug in water is about 1 × 10^−4^ M under studied conditions (Table S1). The stable NPs had a hydrodynamic radius of less than 100 nm in aqueous media and were thermally sensitive, which makes it possible to conclude that TP release from NPs is a result of temperature effects on the structure of NPs and intermolecular bonds between TP molecules and copolymers.

### 3.3. Antioxidant Activity

**Figure 12:**
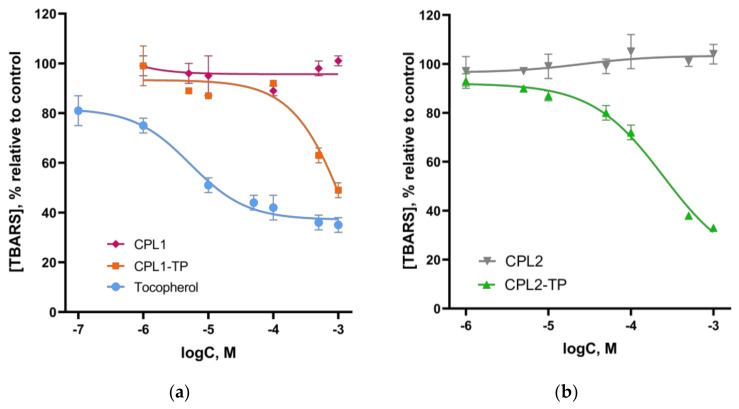
The TBARS accumulation in the mouse brain homogenate in the presence of (**a**) CPL1-TP, CPL1 and tocopherol; (**b**) CPL2 and CPL2-TP. Results are expressed as mean ± SEM as a percentage relative to the control. Control sample—mouse brain homogenate without tested compounds. Tocopherol was dissolved in ethanol. The ethanol content in the sample was 10%, ([TBARS] _ethanol 10%_ = 96 ± 3, *p* > 0.05).

**Figure 13:**
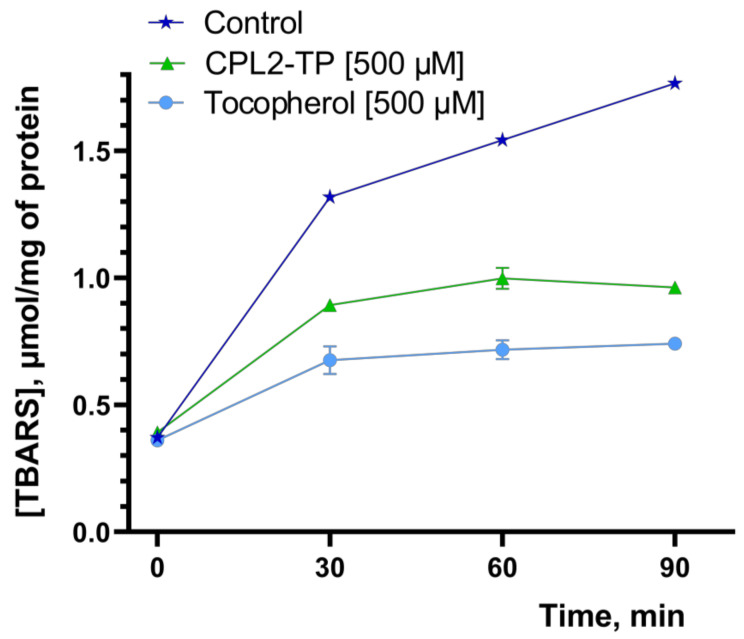
The rate of TBARS cumulation in the mouse brain homogenate in the presence of CPL2-TP and tocopherol (500 µM). Control sample—mouse brain homogenate without tested compounds. Results are shown as mean ± standard deviation.

The antioxidant and ROS scavenging activity were studied by well-known methods: chemiluminescence (CL) and the TBARS. The results are shown in Figure 12 and Figure 13.

#### 3.3.1. TBARS Assay

The effects of obtained polymer compositions with TP, as well as the initial TP and copolymers on the TBARS accumulation (the main of which is malondialdehyde), during spontaneous lipid peroxidation in the mouse brain homogenates were examined. TBARS are formed via the peroxidation of unsaturated fatty acids and proteins, and they are widely used as markers of lipid peroxidation.

Comparison of TBARS accumulation in the homogenate in the presence of CPL1, CPL1-TP, TP, CPL2, CPL2-TP is shown in Figure 12. TBARS accumulation significantly decreased in the presence of CPL1-TP at concentrations 100 µM and above, and CPL2-TP—10 µM and above. At a CPL2-TP concentration of ~1000 µM, the TBARS accumulation (%) was comparable to that of native TP. The concentrations of half-maximal inhibition IC_50_ of the TBARS accumulation process for CPL2-TP and TP were 154 ± 23 and 5.3 ± 0.5 µM, respectively. For CPL1-TP in the concentration range of 1 to 1000 µM, IC_50_ value could not be determined, although the inhibition of lipid peroxidation reached 49% relative to the control. At the same time, the CPL1 and CPL2 copolymers themselves did not affect the accumulation of TBARS products during lipid peroxidation in the mouse brain homogenate. Therefore, the observed effects were due entirely to the TP, which was slowly released from the polymer particles of CPL1 and CPL2, leading to a difference in the percentage of TBARS accumulation.

This is supported by the kinetics of the TBARS accumulation in the presence of CPL2-TP and TP. As seen from the kinetic curves (Figure 13), there was a substantial difference in TBARS cumulation in the presence of tested compounds. TP was more effective than CPL2-TP in inhibiting the LPO process. However, the effectiveness of CPL2-TP for 90 min was also quite high (42 ± 1 vs. 55 ± 1%, respectively, for TP and CPL2-TP relative to the control).

The results obtained on the antioxidant activity of CPL1-TP and CPL2-TP are associated with the release of TP from polymer particles when added to the homogenate. In a previous paper [43], we have shown that incubating NPs loaded by methylpheophorbide a (MPP) as a hydrophobic photosensitizer with liposomes and tissue homogenate leads to a slow increase in the fluorescence of MPP. Most parts of MPP are sent from NPs to biological targets in tissue homogenate within the first 15 min of incubation and achieve a peak by one hour. It can be assumed that TP molecules transfer from NPs to hydrophobic structures such as cell membranes and hydrophobic sites of protein.

#### 3.3.2. Radical Scavenging Capacity in Luminol Chemiluminescence Assay

**Figure 14:**
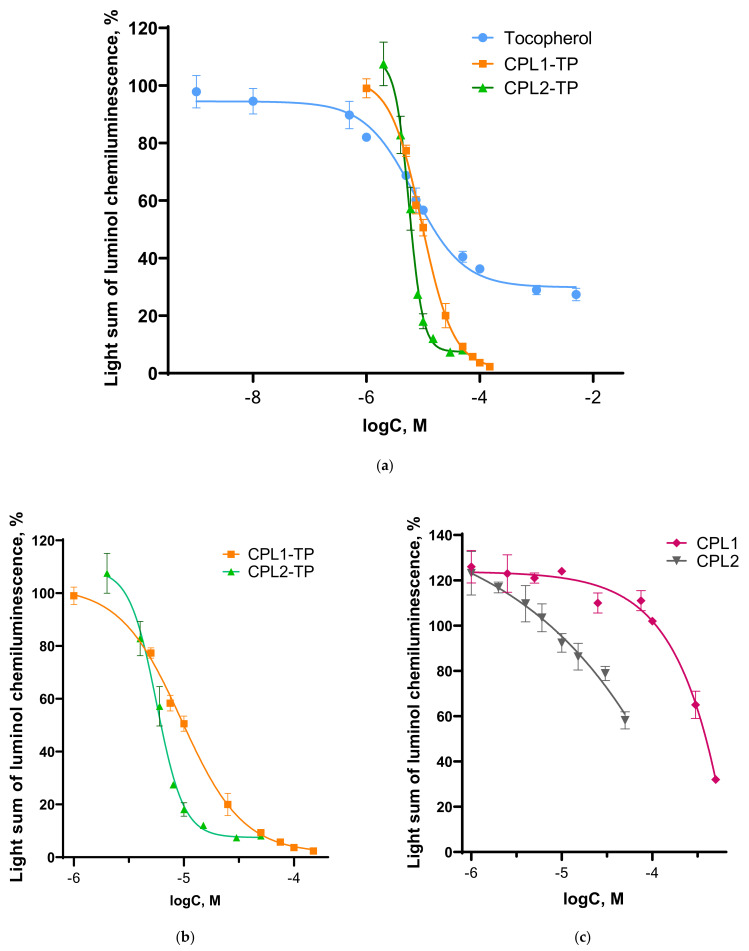
Dose-effect curve of the dependence of the light sum of luminol chemiluminescence on the concentration of tested compounds: (**a**) TP, CPL1-TP, and CPL2-TPl; (**b**) CPL1-TP and CPL2-TP; (**c**) CPL1 and CPL2. CL data are shown as a percentage relative to the control. TP was dissolved in ethanol (1% ethanol in sample). CL light sum for 1% ethanol—110 ± 5% (*p* > 0.05). Control sample—mouse brain homogenate without tested compounds. Results are shown as mean ± standard deviation.

The ability of CPL1-TP, CPL2-TP, initial copolymers of CPL1, CPL2, and native TP to inhibit LPO and show antiradical activity was investigated by luminol chemiluminescence assay. This assay is based on antioxidant-dependent quenching of chemiluminescence generated in mice brain homogenate. Luminol was used to precisely detect hydrogen peroxide (H_2_O_2_), hydroxyl radicals (OH), peroxynitrite (ONOO^−^), and lipid peroxyl radicals [50]. The dependence curves of the chemiluminescence light sum (CL) on the concentration of the studied compounds are shown in Figure 14. IC_50_ values for TP, CPL1-TP, and CPL2-TP were calculated (Table 2).

TP possessed high inhibitory activities for luminol-enhanced CL with IC_50_ value 10.6 ± 1.0 µM (Figure 14a, Table 2). The results indicated that TP inhibited ROS generation during LPO in a dose-dependent manner, which is consistent with the literature [4,62,63]. For both copolymers (Figure 14c), clear inhibition of the luminescence generated from the reaction of radicals and luminol was also observed. For CPL1, this activity was found only at high concentrations (500–300 μM), probably due to copolymers’ aggregation followed by capturing of the luminol. Unlike the copolymer, CPL1-TP at a concentration of 150 μM inhibited the process almost completely (2.3 ± 0.2% relative to the control). The IC_50_ value for CPL1-TP was close to the IC_50_ value of native TP, which indicates the same effective concentration of the antioxidant in the reaction due to TP release from polymer particles.

The CPL2 copolymer reduced the light sum of luminol CL at lower concentrations (15–50 μM). This probably also occurred as a result of the luminol capturing by polymer aggregates. Thus, at a concentration of 50 μM, light sum decreased by 40% relative to the control. However, when the concentration of CPL2 decreased to 10 μM, its effect was not pronounced (*p* > 0.05 from the control according to Student’s *t*-test). As can be seen from Figure 14a,b, in the presence of CPL2-TP, the value of the light sum decreased quite strongly, which indicates a decrease in the level of free radicals in the system. The IC_50_ value for CPL2-TP was almost two times lower than for CPL1-TP and initial TP. This may be due to both the activity of CPL2 and the structure/properties of TP-NPs based on this copolymer and the rate of release of guest molecules; herewith, the effective concentration of TP for LPO inhibition is higher than in the case of CPL1-TP.

The results of ROS inhibitory activity of the tested compounds in mouse brain homogenates using luminol-enhanced CL indicated that encapsulation into amphiphilic copolymers not only increased the solubility of TP, but also maintained its ability to inhibit the LPO.

### 3.4. Antiglycation Activity

**Figure 15:**
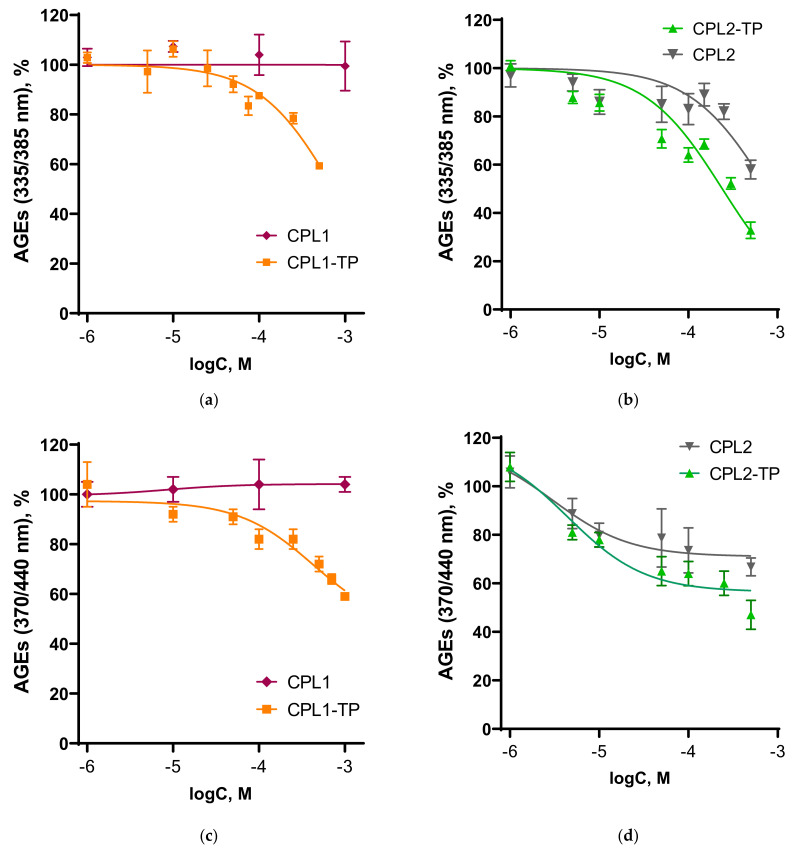
Dose-effect curves for pentosidine-like AGEs (**a**,**b**) and vesperlysines-like AGEs (**c**,**d**) formation in the presence of CPL1, CPL1-TP, CPL2, and CPL2-TP. λ_exc/em_ = 335/385 nm for pentosidine-like AGEs and λ_exc/em_ = 370/440 nm for vesperlysines-like AGEs. Data are presented as value ± SEM.

One of the goals of our work was to estimate the in vitro and anti-AGE activity of water-soluble forms of tocopherol. The effect of tested compounds on the process of non-enzymatic glycation of BSA in vitro was studied by changing the specific fluorescence of advanced glycation end products (AGEs)—vesperlysines-like AGEs and pentosidine-like AGEs, resp., at wavelengths λ_exc/em_ = 370/440 and λ_exc/em_ = 335/385 nm. The dose/response curves are shown in Figure 15.

We observed a decrease in AGE fluorescence in the presence of C1-TPsol, C2-TPsol, and copolymer CPL2 at wavelengths λ_exc/em_ = 370/440 nm, corresponding to the fluorescence of vesperlysines-like AGEs, and λ_exc/em_ = 335/385 nm, corresponding to pentosidine-like AGEs. Consequently, CPL1-TP and CPL2-TP were shown to be glycation inhibitors because the fluorescence of vesperlysines-like AGEs and pentosidine-like AGEs decreases (Figure 15).

CPL1-TP at 1000 µM inhibited formation of vesperlysines-like AGEs by 41 ± 1%, while the initial copolymer was inactive (Figure 15c). CPL2-TP at a concentration of 500 µM inhibited the process by 53 ± 6%, while in the original CPL2 it was by 33 ± 4% (Figure 15d).

CPL2-TP at 500 µM inhibited formation of pentosidine-like AGEs by 67 ± 3% (Figure 15b), and CPL1-TP by 41 ± 1% (Figure 15a). CPL1 copolymer did not affect the fluorescence intensity, while CPL2 reduced it by 42 ± 4%, probably due to the interaction of both the initial BSA and AGEs with this type of copolymer and fluorescence quenching. CPL2-TP inhibited the process by ~50% at concentration of 300 µM. Thus, TP-NPs based on CPL1 and CPL2 inhibited the formation of pentosidine-like AGEs more effectively than vesperlysines-like AGEs. At the same time, CPL2-TP was a more effective inhibitor of protein glycation in vitro than CPL1-TP.

TP dissolved in ethanol has shown antiglycation activity only at its highest concentrations (500–1000 μM) (Supplementary Materials, Figure S1). Thus, 1 and 2% TP in ethanol inhibited glycation by about 50%. In dilute solutions at a TP concentration of 100 μM, antiglycation activity was not observed.

Because of very low solubility of α-tocopherol in pure water and the influence of different types of solvent, IC_50_ values for the glycation process were not obtained. In contrast to the luminol CL and TBARS assay, where a lipid-rich brain homogenate was used, in antiglycation activity assay studies have been done on hydrophilic BSA. Tocopherol dissolved in 100% ethanol reduced AGEs’ formation better at 2% of ethanol in the sample than at 1%. Dissolving of TP in 100% DMSO or increasing the concentration of ethanol in the sample led to activation of the glycation by the solvents themselves. The effects of TP in different solvents are presented in Figure S9 in the Supplementary Materials.

To compare the antiglycation activity of the studied TP-NPs, we used the IC_50_ value of native α-tocopherol, taken from the work of Vinson J.A. et al. (1996) for the glycation reaction of BSA with fructose and glucose (λ_exc/em_ = 350/450 nm), which is 219 µM [64]. We found that CPL2-TP is able to inhibit the glycation process in vitro by about 50% in the concentration range of 300–500 µM, depending on the type of AGEs (vesperlysines-like or pentosidine-like). Thus, there is a slight decrease in the effectiveness of CPL2-TP in inhibiting the formation of AGEs, but the activity remains in a close range of concentrations. At the same time, the data for CPL2-TP, compared with the activity of α-tocopherol, have shown a stronger antiglycation effect of CPL2-TP. This effect can be explained by the good solubility of the copolymer in water, which makes it possible to exhibit an antiglycation effect in vitro (in contrast to α-tocopherol, the antiglycation effect of which has been shown mainly in vivo [13,14,15]), as well as by the direct effect of the CPL2 on the glycation process.

Summarizing the above data, we can conclude that the water-soluble forms of TP obtained in this work based on amphiphilic copolymers (CPL1-TP and CPL2-TP) have inhibitory effect on AGE formation. At the same time, antiglycation activity of CPL2-TP is more pronounced, and can be associated both with the antiglycation activity of the CPL2 itself and the improved properties of the entire complex.

## 4. Conclusions

Water-soluble forms of α-tocopherol were obtained by encapsulating it into particles of amphiphilic copolymers of *N*-vinylpyrrolidone with triethylene glycol dimethacrylate and *N*-vinylpyrrolidone with hexyl methacrylate and triethylene glycol dimethacrylate. NPs of TP have been characterized in aqueous solutions and in the solid state using various physicochemical methods. In water or PBS solution, they showed an absorption band at 290 nm like that of free TP in isopropyl alcohol. The results of quantum chemical modeling show the formation of a hydrogen bond between VP, HMA, and TEGDM units of the copolymer and the OH group of TP.

It has been shown that TP encapsulated in polymer particles has potent antioxidant and antiglycation properties. CPL1-TP and CPL2-TP reduced the formation of TBARS during spontaneous lipid peroxidation in mouse brain homogenate. The effectiveness of the inhibitory action of TP depended on the composition of the initial copolymer. Thus, CPL2-TP based on a ternary copolymer turned out to be a stronger antioxidant than CPL1-TP prepared on the basis of a double copolymer, apparently due to faster release of TP from the CPL2 copolymer upon interaction with biological systems (membranes and hydrophobic sites proteins). However, the inhibitory effect of both polymeric forms of TP was lower than that of the original TP due to delayed release from the polymer particles.

TP encapsulated in CPL1 and CPL2 copolymers inhibits the process of induced LPO that was shown by the luminol-enhanced CL. Compared to native TP, the IC_50_ value decreased twofold for CPL2-TP prepared on the basis of the terpolymer and was the same for CPL1-TP. The initial copolymer contributed to the CPL2-TP activity.

It has been shown that CPL1-TP and CPL2-TP are able to inhibit glycation of bovine serum albumin in vitro and suppress the formation of vesperlysines-like AGEs and pentosidine-like AGEs. At the same time, CPL1-TP and CPL2-TP inhibit the formation of pentosidine-like AGEs more effectively than vesperlysines-like AGEs. In general, CPL2-TP is a stronger inhibitor of protein glycation in vitro than CPL1-TP. The water solubility and amphiphilicity of the obtained compounds increases the bioavailability of TP, and the presence of pronounced antioxidant and antiglycation properties makes it possible to consider them as promising forms of TP for biomedical applications.

## Figures and Tables

**Table 1:** Physicochemical characteristics of the CPL1 and CPL2.

Copolymers	[VP]:[(di)methacrylate] /mol%	M_w_, kDa	PD	CAC in Water/ mg mL^−1^	R_h_^a^ in Water/nm 25 °C
CPL1 CPL2	95.3:4.7 96.6:3.4	34.9 33.7	1.3 1.3	2.8 0.3	4.4 ± 2.9; 77.2 ± 37.4 89.9 ± 14.5

^a^ Measured at a copolymer concentration in water of 2.5 mg mL^−1^.

**Table 2:** Half-maximal inhibitory concentrations (IC_50_) of tert-butyl hydroperoxide induced luminol chemiluminescence in mouse brain homogenate in the presence of TP, CPL1-TP, and CPL2-TP.

Compound	CPL1-TP	CPL2-TP	TP
IC_50_, µM	9.7 ± 0.7	5.1 ± 0.5	10.6 ± 1.0

## Data Availability

Not applicable.

## Supplementary Materials

The following supporting information can be downloaded at: https://www.mdpi.com/article/10.3390/pharmaceutics15051388/s1, Table S1. Concentrations of reagents and conditions for TP encapsulation in CPL1. Table S2. Concentrations of reagents and conditions for TP encapsulation in CPL2. Table S3. Concentrations of reagents and conditions for TP encapsulation in CPL2. Figure S1. Intensity of light scattering distribution curves by IPA solution of CPL1 and CPL2 at 25 °C. The copolymer concentrations were 7 mg mL^−1^. Figure S2. Absorption spectra of solutions of TP in IPA. Cuvettes were 0.2 or 1 cm. Figure S3. Absorption spectra of aqueous buffer solution in control experiments performed without a copolymer. Cuvette was 1 cm. Figure S4. Absorption spectra of CPL1-TP in aqueous buffer solution (pH 7.2–7.4), the curves correspond to the data in Table S1. The spectrum of the water buffer is shown with a dotted line. Cuvette was 1 cm. Figure S5. Absorption spectra (a) of CPL2-TP in aqueous buffer solution (pH 7.2–7.4). Cuvette was 1 cm; curves correspond to the data in Table S2 (the spectrum of PBS is shown with a dotted line); and dependence of the optical density of the absorption band of TP on the initial concentration of TP (b) in the coordinates of the Connors equation. Figure S6. Intensity of light scattering distribution curves of CPL1-TP in PBS over the hydrodynamic radii of scattering centers in the temperature range of 25–47 °C. [CPL1-TP] = 3 mg/mL. Figure S7. Dependencies of the light scattering intensity (a) and hydrodynamic radius (b) of scattering centers of CPL1-TP and the initial CPL1 on temperature. Figure S8. Absorption spectra of freshly prepared aqueous buffer solutions of CPL2-TP (solid line) and after storage for 3 days at room temperature (dotted line). Spectra correspond to the data in Table S3. Figure S9. Effect of α-tocopherol and solvents on vesperlysines-like AGEs (a) and pentosidine-like AGEs (b) formation during BSA glycation. λ_exc/em_ = 370/440 nm for vesperlysines-like AGEs and λ_exc/em_ = 335/385 nm for pentosidine-like AGEs. Data are presented as value ± SEM.

## References

[1] Liu P., Feng Y., Wang Y., Zhou Y., Zhao L. (2015). Protective Effect of Vitamin E against Acute Kidney Injury. Biomed. Mater. Eng..

[2] Song Q., Liu J., Dong L., Wang X., Zhang X. (2021). Novel Advances in Inhibiting Advanced Glycation End Product Formation Using Natural Compounds. Biomed. Pharmacother..

[3] Niki E., Traber M.G. (2012). A History of Vitamin E. Ann. Nutr. Metab..

[4] Niki E. (2021). Lipid Oxidation That Is, and Is Not, Inhibited by Vitamin E: Consideration about Physiological Functions of Vitamin E. Free Radic. Biol. Med..

[5] Singh P., Jayaramaiah R.H., Agawane S.B., Vannuruswamy G., Korwar A.M., Anand A., Dhaygude V.S., Shaikh M.L., Joshi R.S., Boppana R. (2016). Potential Dual Role of Eugenol in Inhibiting Advanced Glycation End Products in Diabetes: Proteomic and Mechanistic Insights. Sci. Rep..

[6] Chang C.-J., Hsien R.-H., Wang H.-F., Chin M.-Y., Huang S.-Y. (2005). Effects of Glucose and α-Tocopherol on Low-Density Lipoprotein Oxidation and Glycation. Ann. N. Y. Acad. Sci..

[7] Pazdro R., Burgess J.R. (2012). Differential Effects of α-Tocopherol and N-Acetyl-Cysteine on Advanced Glycation End Product-Induced Oxidative Damage and Neurite Degeneration in SH-SY5Y Cells. Biochim. Biophys. Acta Mol. Basis Dis..

[8] Niki E., Noguchi N. (2004). Dynamics of Antioxidant Action of Vitamin E. Acc. Chem. Res..

[9] Ihara Y., Yamada Y., Toyokuni S., Miyawaki K., Ban N., Adachi T., Kuroe A., Iwakura T., Kubota A., Hiai H. (2000). Antioxidant α-Tocopherol Ameliorates Glycemic Control of GK Rats, a Model of Type 2 Diabetes. FEBS Lett..

[10] Je H.D., Shin C.Y., Park H.S., Huh I.H., Sohn U.D. (2001). The Comparison of Vitamin C and Vitamin E on the Protein Oxidation of Diabetic Rats. J. Auton. Pharmacol..

[11] Minamiyama Y., Takemura S., Bito Y., Shinkawa H., Tsukioka T., Nakahira A., Suehiro S., Okada S. (2008). Supplementation of α -Tocopherol Improves Cardiovascular Risk Factors via the Insulin Signalling Pathway and Reduction of Mitochondrial Reactive Oxygen Species in Type II Diabetic Rats. Free Radic. Res..

[12] Pazdro R., Burgess J.R. (2010). The Role of Vitamin E and Oxidative Stress in Diabetes Complications. Mech. Ageing Dev..

[13] Rafraf M., Bazyun B., Sarabchian M.A., Safaeiyan A., Gargari B.P. (2016). Vitamin E Improves Serum Paraoxonase-1 Activity and Some Metabolic Factors in Patients with Type 2 Diabetes: No Effects on Nitrite/Nitrate Levels. J. Am. Coll. Nutr..

[14] Manzella D., Barbieri M., Ragno E., Paolisso G. (2001). Chronic Administration of Pharmacologic Doses of Vitamin E Improves the Cardiac Autonomic Nervous System in Patients with Type 2 Diabetes. Am. J. Clin. Nutr..

[15] Fardoun R.Z. (2007). The Use of Vitamin E in Type 2 Diabetes Mellitus. Clin. Exp. Hypertens..

[16] Selvaraj N., Bobby Z., Sathiyapriya V. (2006). Effect of Lipid Peroxides and Antioxidants on Glycation of Hemoglobin: An In Vitro Study on Human Erythrocytes. Clin. Chim. Acta.

[17] Schleicher E.D., Wagner E., Nerlich A.G. (1997). Increased Accumulation of the Glycoxidation Product N(Epsilon)-(Carboxymethyl)Lysine in Human Tissues in Diabetes and Aging. J. Clin. Investig..

[18] Traber M.G. (2013). Vitamin E: Metabolism and Requirements. Encyclopedia of Human Nutrition.

[19] Tarugi P., Averna M. (2011). Hypobetalipoproteinemia: Genetics, Biochemistry, and Clinical Spectrum. Adv. Clin. Chem..

[20] Cuerq C., Henin E., Restier L., Blond E., Drai J., Marçais C., Di Filippo M., Laveille C., Michalski M.-C., Poinsot P. (2018). Efficacy of Two Vitamin E Formulations in Patients with Abetalipoproteinemia and Chylomicron Retention Disease. J. Lipid Res..

[21] Singh P., Wu L., Ren X., Zhang W., Tang Y., Chen Y., Carrier A., Zhang X., Zhang J. (2020). Hyaluronic-Acid-Based β-Cyclodextrin Grafted Copolymers as Biocompatible Supramolecular Hosts to Enhance the Water Solubility of Tocopherol. Int. J. Pharm..

[22] Guo Y., Luo J., Tan S., Otieno B.O., Zhang Z. (2013). The Applications of Vitamin E TPGS in Drug Delivery. Eur. J. Pharm. Sci..

[23] Chen Y., Feng S., Liu W., Yuan Z., Yin P., Gao F. (2017). Vitamin E Succinate-Grafted-Chitosan Oligosaccharide/RGD-Conjugated TPGS Mixed Micelles Loaded with Paclitaxel for U87MG Tumor Therapy. Mol. Pharm..

[24] Gao M., Deng J., Chu H., Tang Y., Wang Z., Zhao Y., Li G. (2017). Stereoselective Stabilization of Polymeric Vitamin E Conjugate Micelles. Biomacromolecules.

[25] Hobson R. (2016). Vitamin E and Wound Healing: An Evidence-Based Review. Int. Wound J..

[26] Zuccari G., Alfei S., Zorzoli A., Marimpietri D., Turrini F., Baldassari S., Marchitto L., Caviglioli G. (2021). Increased Water-Solubility and Maintained Antioxidant Power of Resveratrol by Its Encapsulation in Vitamin E TPGS Micelles: A Potential Nutritional Supplement for Chronic Liver Disease. Pharmaceutics.

[27] Mu L., Feng S. (2003). A Novel Controlled Release Formulation for the Anticancer Drug Paclitaxel (Taxol^®^): PLGA Nanoparticles Containing Vitamin E TPGS. J. Control. Release.

[28] Sheng X., Fan L., He C., Zhang K., Mo X., Wang H. (2013). Vitamin E-Loaded Silk Fibroin Nanofibrous Mats Fabricated by Green Process for Skin Care Application. Int. J. Biol. Macromol..

[29] Bramley P., Elmadfa I., Kafatos A., Kelly F., Manios Y., Roxborough H., Schuch W., Sheehy P., Wagner K.-H. (2000). Vitamin E. J. Sci. Food Agric..

[30] Yang Y., McClements D.J. (2013). Vitamin E Bioaccessibility: Influence of Carrier Oil Type on Digestion and Release of Emulsified α-Tocopherol Acetate. Food Chem..

[31] García P., Vega J., Jimenez P., Santos J., Robert P. (2013). Alpha-tocopherol Microspheres with Cross-linked and Acetylated Inulin and Their Release Profile in a Hydrophilic Model. Eur. J. Lipid Sci. Technol..

[32] Cheng C.-C., Chang F.-C., Kao W.-Y., Hwang S.-M., Liao L.-C., Chang Y.-J., Liang M.-C., Chen J.-K., Lee D.-J. (2016). Highly Efficient Drug Delivery Systems Based on Functional Supramolecular Polymers: In Vitro Evaluation. Acta Biomater..

[33] Jain H., Chella N. (2021). Methods to Improve the Solubility of Therapeutical Natural Products: A Review. Environ. Chem. Lett..

[34] Zhang X., Xing H., Zhao Y., Ma Z. (2018). Pharmaceutical Dispersion Techniques for Dissolution and Bioavailability Enhancement of Poorly Water-Soluble Drugs. Pharmaceutics.

[35] Dima Ş., Dima C., Iordăchescu G. (2015). Encapsulation of Functional Lipophilic Food and Drug Biocomponents. Food Eng. Rev..

[36] Torchilin V. (2006). Introduction. Nanocarriers for Drug Delivery: Needs and Requirements. Nanoparticulates as Drug Carriers.

[37] Kwon G.S., Forrest M.L. (2006). Amphiphilic Block Copolymer Micelles for Nanoscale Drug Delivery. Drug Dev. Res..

[38] Letchford K., Burt H. (2007). A Review of the Formation and Classification of Amphiphilic Block Copolymer Nanoparticulate Structures: Micelles, Nanospheres, Nanocapsules and Polymersomes. Eur. J. Pharm. Biopharm..

[39] Kurmaz S.V., Fadeeva N.V., Komendant A.V., Ignatiev V.M., Emelyanova N.S., Shilov G.V., Stupina T.S., Filatova N.V., Lapshina M.A., Terentyev A.A. (2022). New Amphiphilic Terpolymers of N-Vinylpyrrolidone with Poly(Ethylene Glycol) Methyl Ether Methacrylate and Triethylene Glycol Dimethacrylate as Carriers of the Hydrophobic Fluorescent Dye. Polym. Bull..

[40] Kurmaz S.V., Rudneva T.N., Sanina N.A. (2018). New Nitric Oxide-Carrier Systems Based on an Amphiphilic Copolymer of N -Vinylpyrrolidone with Triethylene Glycol Dimethacrylate. Mendeleev Commun..

[41] Faingol’d I.I., Lozhkin A.D., Smolina A.V., Soldatova Y.V., Obraztsova N.A., Kurmaz S.V., Romanova V.S., Shtol’ko V.N., Kotel’nikova R.A. (2018). Membranotropic Properties of Fullerene-Containing Amphiphilic (Co)Polymers of N-Vinylpyrrolidone. Russ. Chem. Bull..

[42] Kurmaz S.V., Fadeeva N.V., Fedorov B.S., Kozub G.I., Emel’yanova N.S., Kurmaz V.A., Manzhos R.A., Balakina A.A., Terentyev A.A. (2020). New Antitumor Hybrid Materials Based on PtIV Organic Complex and Polymer Nanoparticles Consisting of N-Vinylpyrrolidone and (Di)Methacrylates. Mendeleev Commun..

[43] Rybkin A.Y., Kurmaz S.V., Urakova E.A., Filatova N.V., Sizov L.R., Kozlov A.V., Koifman M.O., Goryachev N.S. (2023). Nanoparticles of N-Vinylpyrrolidone Amphiphilic Copolymers and Pheophorbide a as Promising Photosensitizers for Photodynamic Therapy: Design, Properties and In Vitro Phototoxic Activity. Pharmaceutics.

[44] Kurmaz S.V., Obraztsova N.A., Balakina A.A., Terent’ev A.A. (2016). Preparation of the Amphiphilic Copolymer of N-Vinylpyrrolidone with Triethylene Glycol Dimethacrylate Nanoparticles and the Study of Their Properties In Vitro. Russ. Chem. Bull..

[45] Kurmaz S.V., Fadeeva N.V., Terentiev A.A. (2020). Method for Obtaining Nanoscale Systems of Low Molecular Weight Biologically Active Compounds Based on Amphiphilic Copolymers of N-Vinylpyrrolidone with Branched (Di)Methacrylates for Cosmeceutical Applications. RF Patent.

[46] Connors K.A. (1987). Binding Constants: The Measurement of Molecular Complex Stability.

[47] Todd A., Keith T. (2010). Gristmill Software.

[48] Espinosa E., Molins E., Lecomte C. (1998). Hydrogen Bond Strengths Revealed by Topological Analyses of Experimentally Observed Electron Densities. Chem. Phys. Lett..

[49] Lowry O., Rosebrough N.J., Farr A.L., Randall R.J. (1951). Protein Measurement with the Folin Phenol Reagent. Biol. Chem..

[50] Poletaeva D.A., Soldatova Y.V., Smolina A.V., Savushkin M.A., Klimanova E.N., Sanina N.A., Faingold I.I. (2022). The Influence of Cationic Nitrosyl Iron Complex with Penicillamine Ligands on Model Membranes, Membrane-Bound Enzymes and Lipid Peroxidation. Membranes.

[51] Soldatova Y.V., Zhilenkov A.V., Kraevaya O.A., Troshin P.A., Faingold I.I., Kotelnikova R.A. (2022). Antioxidant and Antiglycation Activity of Pentaamino Acid Derivatives of C60 Fullerene. Nanotechnol. Russ. (Nanobiotechnol. Rep.).

[52] Chen Y.F., Roan H.Y., Lii C.K., Huang Y.C., Wang T.-S. (2011). Relationship between Antioxidant and Antiglycation Ability of Saponins, Polyphenols, and Polysaccharides in Chinese Herbal Medicines Used to Treat Diabetes. J. Med. Plants Res..

[53] Matsuura N., Aradate T., Sasaki C., Kojima H., Ohara M., Hasegawa J., Ubukata M. (2002). Screening System for the Maillard Reaction Inhibitor from Natural Product Extracts. J. Health Sci..

[54] Séro L., Sanguinet L., Blanchard P., Dang B., Morel S., Richomme P., Séraphin D., Derbré S. (2013). Tuning a 96-Well Microtiter Plate Fluorescence-Based Assay to Identify AGE Inhibitors in Crude Plant Extracts. Molecules.

[55] Kurmaz S.V., Ignatiev V.M., Emel’yanova N.S., Kurmaz V.A., Konev D.V., Balakina A.A., Terentyev A.A. (2022). New Nanosized Systems Doxorubicin—Amphiphilic Copolymers of N-Vinylpyrrolidone and (Di)Methacrylates with Antitumor Activity. Pharmaceutics.

[56] Kurmaz S.V., Pyryaev A.N. (2010). Synthesis of N-Vinyl-2-Pyrrolidone-Based Branched Copolymers via Crosslinking Free-Radical Copolymerization in the Presence of a Chain-Transfer Agent. Polym. Sci. Ser. B.

[57] Kurmaz S.V., Fadeeva N.V., Ignat’ev V.M., Kurmaz V.A., Kurochkin S.A., Emel’yanova N.S. (2020). Structure and State of Water in Branched N-Vinylpyrrolidone Copolymers as Carriers of a Hydrophilic Biologically Active Compound. Molecules.

[58] Zhou Y., Huang W., Liu J., Zhu X., Yan D. (2010). Self-Assembly of Hyperbranched Polymers and Its Biomedical Applications. Adv. Mater..

[59] Kurmaz S.V., Fadeeva N.V., Skripets J.A., Komendant R.I., Ignatiev V.M., Emel’yanova N.S., Soldatova Y.V., Faingold I.I., Poletaeva D.A., Kotelnikova R.A. (2022). New Water-Soluble Forms of α-Tocopherol: Preparation and Study of Antioxidant Activity In Vitro. Mendeleev Commun..

[60] Checheta O.V., Safonova E.F., Slivkin A.I. (2010). Study of Hydrogen Bonds of α-Tocopherol IR Spectroscopy Method. Vestn. VGU.

[61] Nadirov N.K., Nadirov N.K., Emanuel N.M., Evstigneeva R.P. (1991). Tocopherols and Their Use in Medicine and Agriculture.

[62] Niki E. (2018). Oxidant-Specific Biomarkers of Oxidative Stress. Association with Atherosclerosis and Implication for Antioxidant Effects. Free Radic. Biol. Med..

[63] Traber M.G. (2007). Vitamin E Regulatory Mechanisms. Annu. Rev. Nutr..

[64] Vinson J.A., Howard T.B. (1996). Inhibition of Protein Glycation and Advanced Glycation End Products by Ascorbic Acid and Other Vitamins and Nutrients. J. Nutr. Biochem..

