# Supplementary material for: Design and Investigation of New Water-Soluble Forms of α-Tocopherol with Antioxidant and Antiglycation Activity Using Amphiphilic Copolymers of N-Vinylpyrrolidone
Source: Pharmaceutics. 2023 Apr 30;15(5):1388. doi: 10.3390/pharmaceutics15051388 (PMC10223339; doi:10.3390/pharmaceutics15051388)
Supplement: Supplementary file 1 [file pharmaceutics-15-01388-s001.zip › pharmaceutics-2332847-supplementary.pdf]

Supplementary Materials: **Design and investigation of new water-soluble forms of α-tocopherol with antioxidant and antiglycation activity using amphiphilic copolymers of *N*-vinylpyrrolidone**

Yuliya V. Soldatova[1*], Irina I. Faingold[1], Darya A. Poletaeva[1], Alexei V. Kozlov[1], Nina S. Emel′yanova[1], Igor I. Khodos[1], Dmitry A. Chernyaev[1], Svetlana V. Kurmaz[1]

Table S1. Concentrations of reagents and conditions for TP encapsulation in CPL1

[CPL1] in IPA/mg mL^{-1}	[TP] in IPA/mg mL^{-1}	Volume ratios of solutions copolymer and TP/mL	TP content per CPL1/%	[CPL1] in water buffer/mg mL^{-1}	[TP]×10^4 in water buffer/M
0.35	0.057	4 : 0	0	0.175	0
		4 : 0.3	1.2		0.1
		4 : 0.6	2.4		0.2
		4 : 1.2	4.8		0.4
		4 : 1.8	7.3		0.6
		4 : 2.4	9.6		0.8

Table S2. Concentrations of reagents and conditions for TP encapsulation in CPL2

[CPL2] in IPA/ mg ml^{-1}	[TP] in IPA/mg mL^{-1}	Volume ratios of solutions copolymer and TP/mL	TP content per CPL2/%	[CPL2] in water buffer/mg mL^{-1}	[TP] ×10^5 in water buffer/M
		4:0	0		0
		4:0.05	1.5		0.6
0.17	0.21	4:0.1	3.0	0.085	1.2
		4:0.15	4.5		1.8
		4:0.2	6.0		2.4
		4:0.25	7.5		3.0
		4:0.3	9.0		3.7

Table S3. Concentrations of reagents and conditions for TP encapsulation in CPL2

[CPL2] in IPA/ mg mL^{-1}	[TP] in IPA/mg mL^{-1}	Volume ratios of solutions copolymer and TP/mL	TP content per CPL2/%	[CPL2] in water buffer/mg mL^{-1}	[TP] ×10^5 in water buffer/M
		4:0	0		0
		4:0.05	3.5		1.4
0.17	0.47	4:0.1	6.9	0.085	2.7
		4:0.15	10.4		4.1
		4:0.2	13.8		5.5

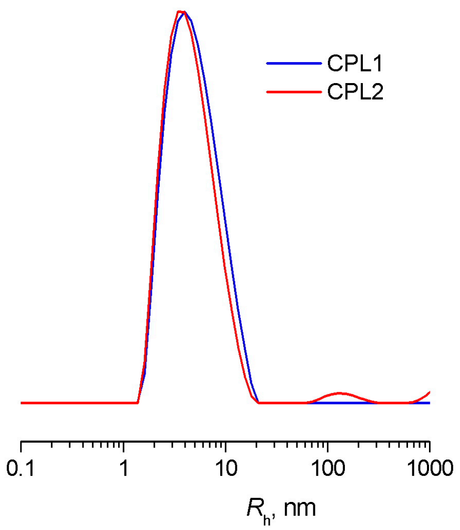

Figure S1. Intensity of light scattering distribution curves by IPA solution of CPL1 and CPL2 at 25 °C. The copolymers concentrations were 7 mg mL^{-1}.

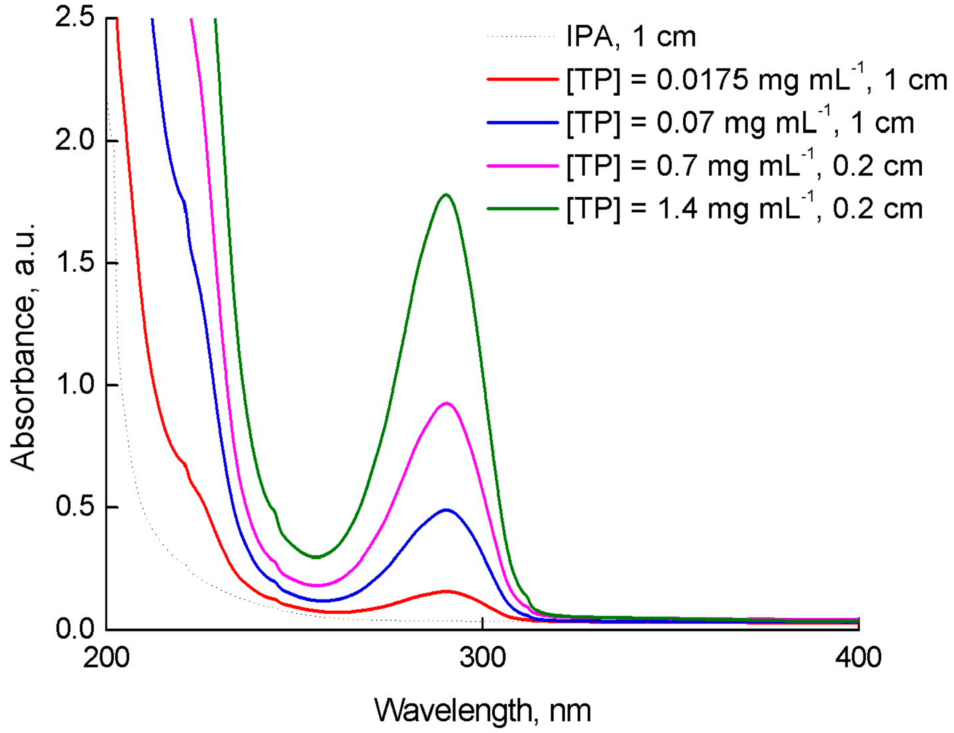

Figure S2. Absorption spectra of TP solutions in IPA. Cuvettes were 0.2 or 1 cm.

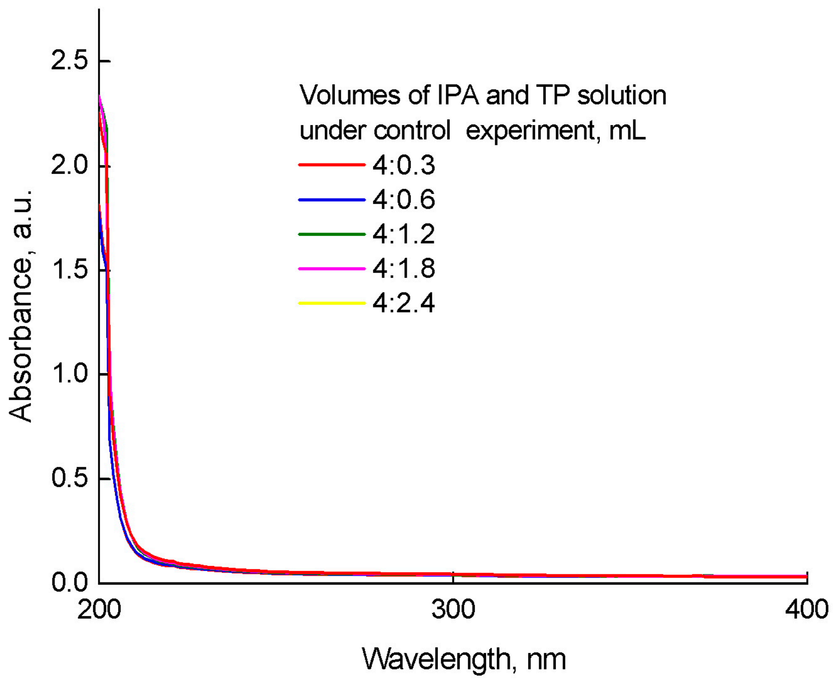

Figure S3. Absorption spectra of aqueous buffer solution in control experiments performed without a copolymer. Cuvette is 1 cm.

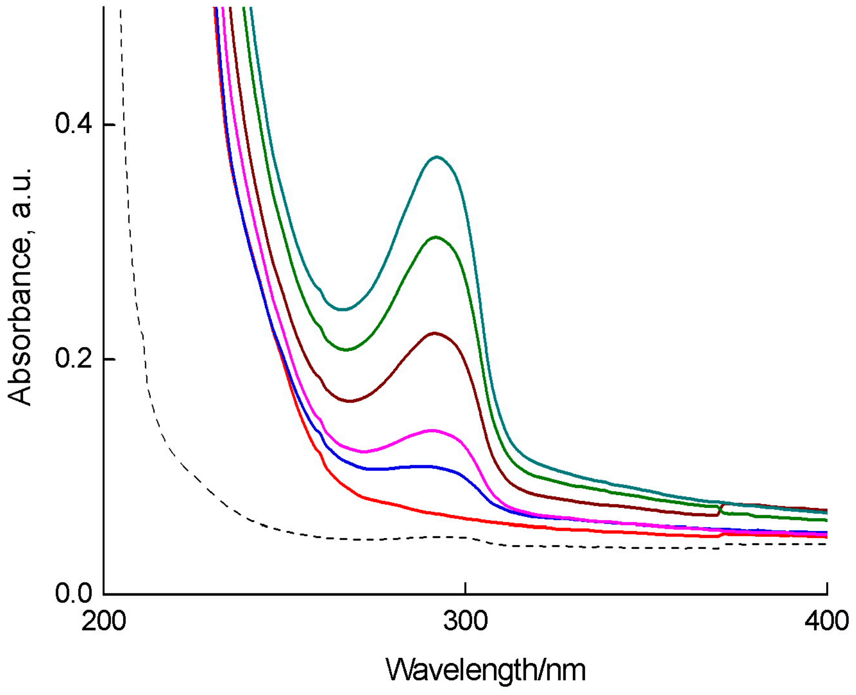

Figure S4. Absorption spectra of CPL1-TP in aqueous buffer solution (pH 7.2-7.4), the curves correspond to the data in Table S1. The spectrum of the water buffer is shown with a dotted line. Cuvette is 1 cm.

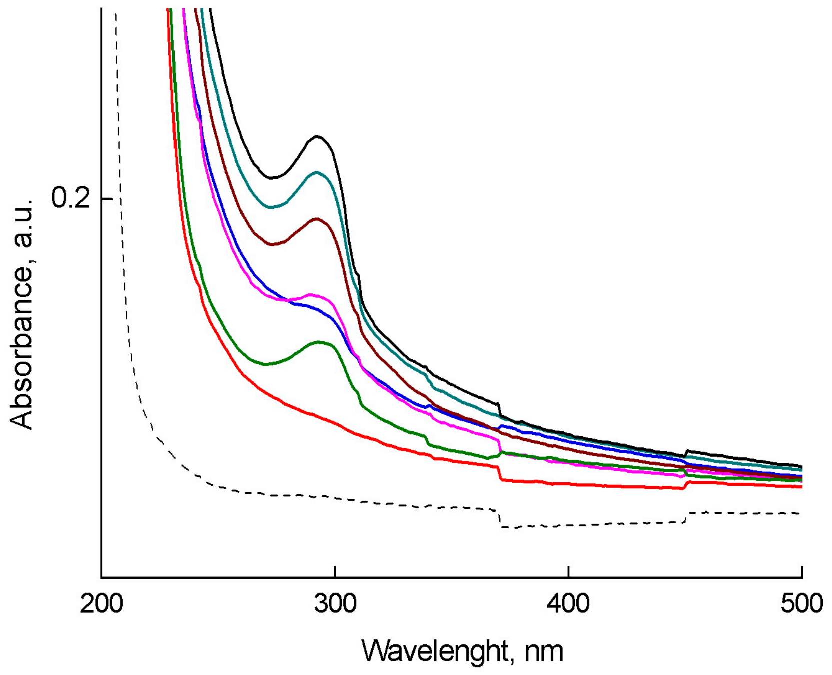

(*a*)

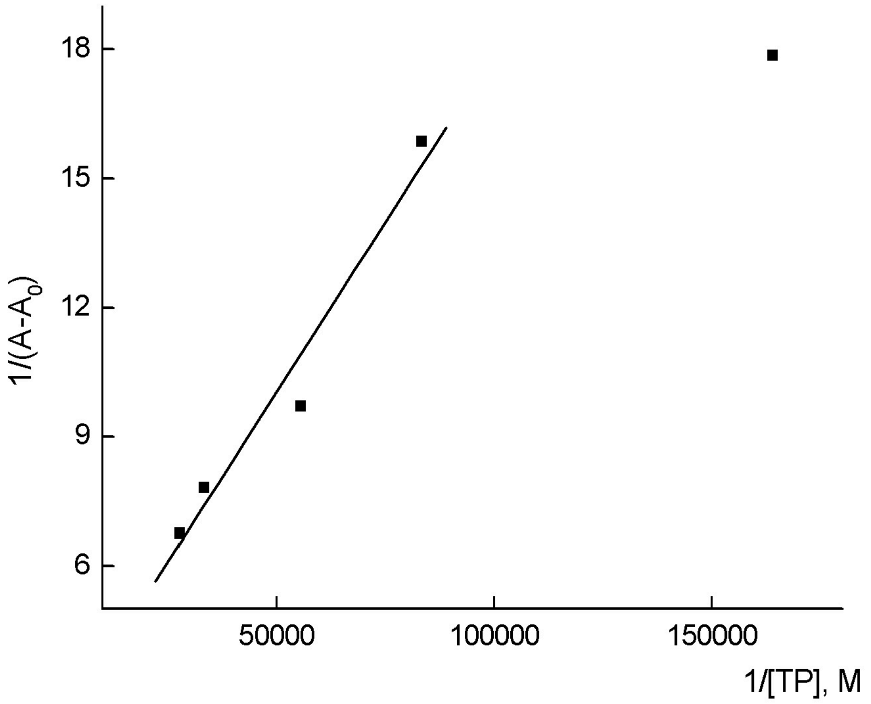

(*b*)

Figure S5. Absorption spectra (*a*) of CPL2-TP in aqueous buffer solution (pH 7.2-7.4), cuvette is 1 cm; curves correspond to the data in Table S2 (the spectrum of PBS is shown with a dotted line); and dependence of the optical density of the absorption band of TP on the initial concentration of TP (*b*) in the coordinates of the Connors equation.

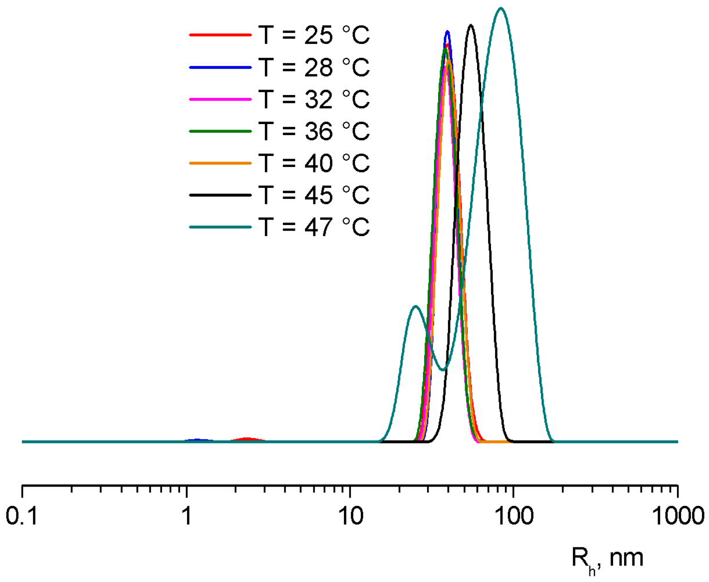

Figure S6. Intensity of light scattering distribution curves of CPL1-TP in PBS over the hydrodynamic radii of scattering centers in the temperature range of 25-47 °C. [CPL1-TP] = 3 mg/mL.

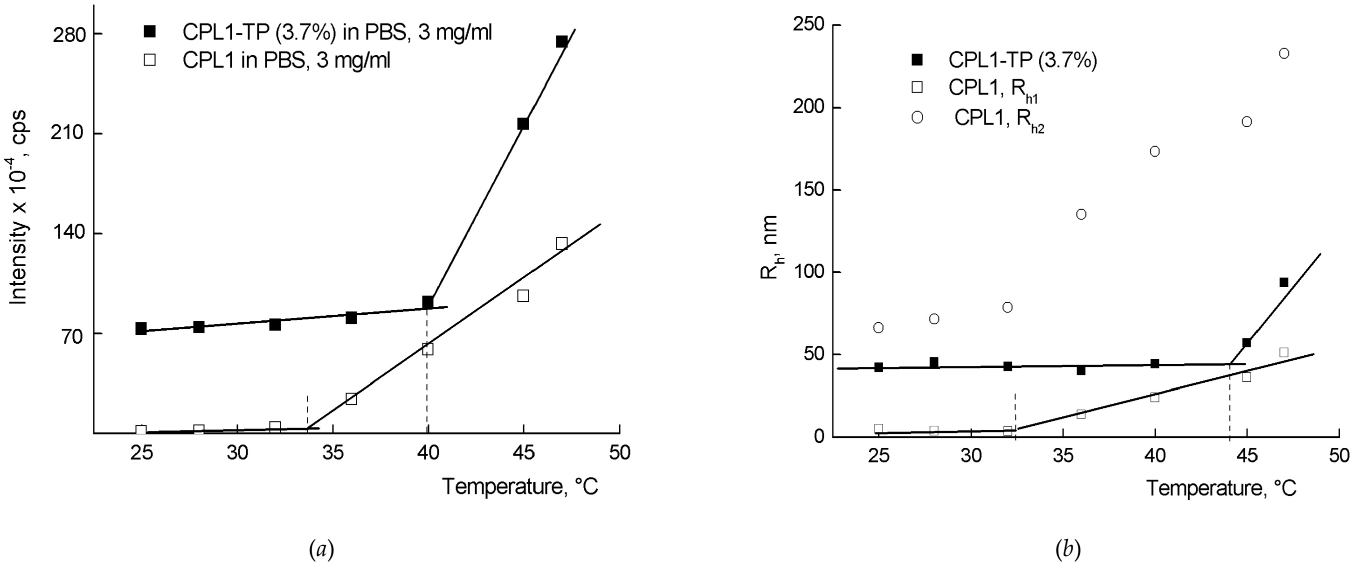

Figure S7. Dependencies of the light scattering intensity (*a*) and hydrodynamic radius (*b*) of scattering centers of CPL1-TP and the initial CPL1 on temperature.

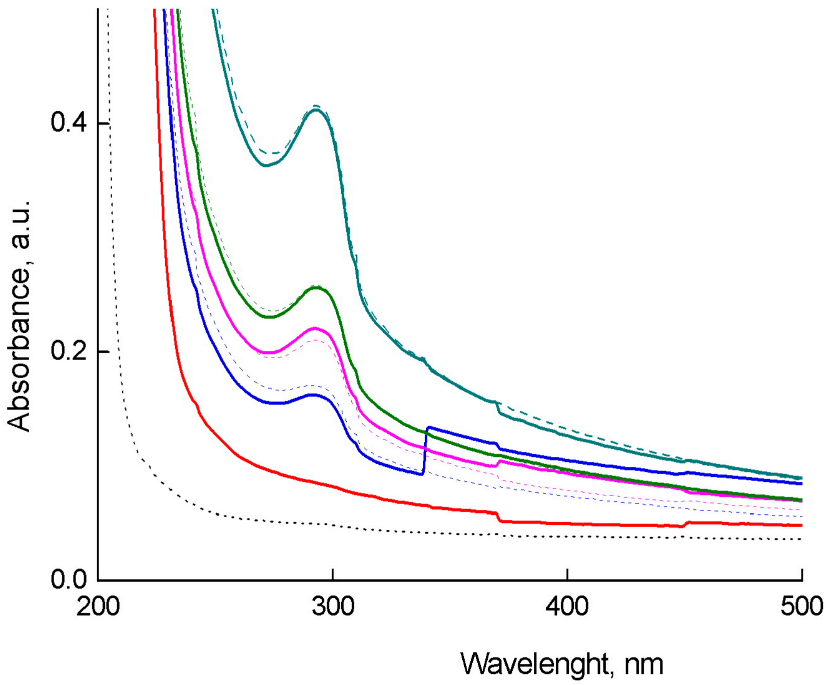

Figure S8. Absorption spectra of freshly prepared aqueous buffer solutions of CPL2-TP (solid line) and after storage for 3 days at room temperature (dotted line). Spectra correspond to the data in Table S3.

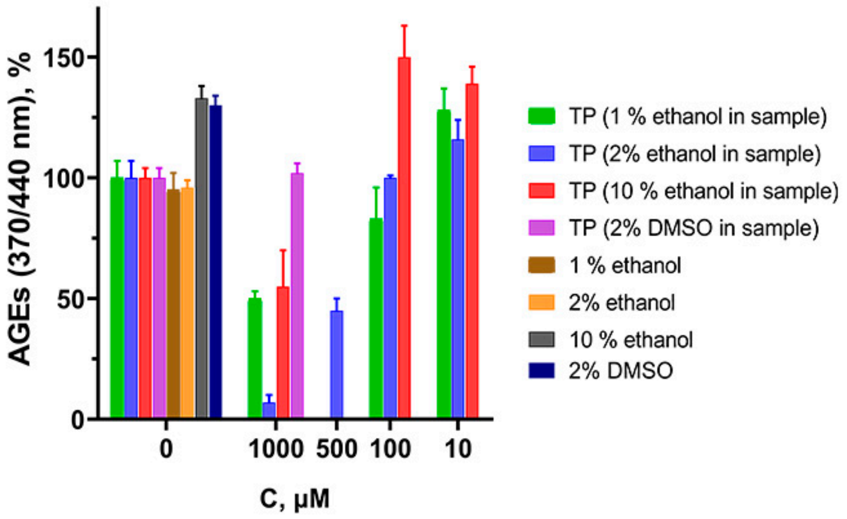

(a)

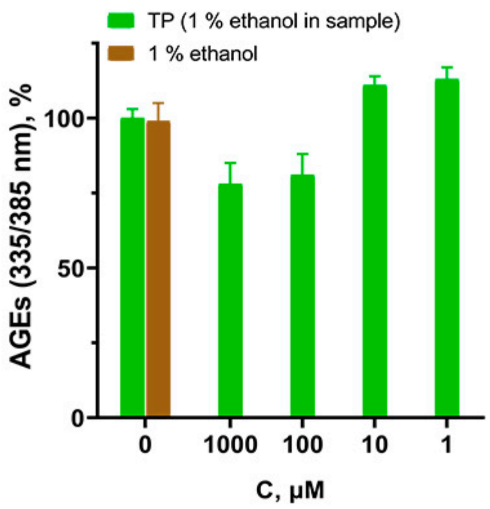

(b)

Figure S9. Effect of α-tocopherol and solvents on vesperlysines-like AGEs (a) and pentosidine-like AGEs (b) formation during BSA glycation. $\lambda_{exc/em}$=370/440 nm for vesperlysines-like AGEs and $\lambda_{exc/em}$ = 335/385 nm for pentosidine-like AGEs. Data are presented as value ± SEM.
